# Plant neighbours, not consumers, drive intraspecific phytochemical changes of two grassland species in a field experiment

**DOI:** 10.1093/aobpla/plaf071

**Published:** 2025-12-15

**Authors:** Joshua I Brian, Adrien Le Guennec, Elizabeth T Borer, Eric W Seabloom, Michael A Chadwick, Jane A Catford

**Affiliations:** Department of Geography, King’s College London, 40 Aldwych, London WC2B 4BG, United Kingdom; Centre for Biomolecular Spectroscopy, King’s College London, New Hunt's House, London SE1 1UL, United Kingdom; Department of Ecology, Evolution and Behavior, University of Minnesota, 1479 Gortner Ave, Saint Paul, MN 55108, United States; Department of Ecology, Evolution and Behavior, University of Minnesota, 1479 Gortner Ave, Saint Paul, MN 55108, United States; Department of Geography, King’s College London, 40 Aldwych, London WC2B 4BG, United Kingdom; Department of Geography, King’s College London, 40 Aldwych, London WC2B 4BG, United Kingdom; Fenner School of Environment & Society, The Australian National University, 141 Linnaeus Way, Canberra, ACT 2601, Australia; Populations & Communities

**Keywords:** Cedar Creek Ecosystem Science Reserve, trade-off, competition, diversity, insect herbivore, fungal pathogen

## Abstract

Plants use chemicals to respond to their environments. Despite the impact of competition on plant productivity, few studies consider how plant–plant competition affects phytochemistry; most phytochemistry studies focus on plant–consumer interactions. It therefore remains unclear how plants chemically respond to changes in both competition and consumer pressure. We used ^1^H-NMR spectroscopy to characterize the phytochemistry (both primary and secondary metabolites) of a C4 grass (*Andropogon gerardi*) and a legume (*Lespedeza capitata*) in a field experiment. Both species were grown with intraspecific or interspecific neighbours (monoculture or 16-species polyculture) with or without a combined fungicide + insecticide treatment (consumers reduced vs. consumers present) in a factorial design. We measured species aboveground biomass, healthy plant cover (NDVI) and phytochemistry in the four treatments to determine whether plants alter their biomass, phytochemistry, or both in response to neighbours and herbivory. Phytochemistry of *A. gerardi* did not vary with neighbour identity or consumers, in contrast to *A. gerardi* biomass, which was higher under interspecific competition and when consumers were reduced. Phytochemistry of *L. capitata* was also unrelated to consumer reduction, though *L. capitata* had higher NDVI under reduced consumers. However, *L. capitata* had lower biomass and exhibited phytochemical signs of metabolic stress (lower sugars and higher amino acid production) when grown with interspecific neighbours. Theory and empirical work have focused on coevolution with consumers as driving phytochemical variation, but our results suggest that—at community scales—the competitive environment may be more important than consumer pressure in determining short-term phytochemical responses of some species.

## Teaser

Plants use chemicals to respond to consumers, competitors, and the wider environment. Most work has focused on consumers in driving the diversity of these chemicals, but less clear is how plants change their chemical investment in response to competing stressors under real-world conditions. We show that at small scales, plant neighbours rather than consumers are more important in determining chemical investment, but this effect varies by species. Future work needs to consider competitors alongside consumers as key drivers of local phytochemical composition.

## Introduction

Plants produce a high diversity of primary and secondary metabolites, collectively ‘phytochemicals’ (Jones and Firn 1991, Endara et al. 2023). Plants use these chemicals to respond to consumers (Agrawal et al. 2012, Richards et al. 2015), mutualists (Bruce and Pickett 2011), competitors (Bais et al. 2006), and environmental stressors (Zobayed et al. 2007), which can all interact (Cipollini 2004, Schuman and Baldwin 2016). Phytochemistry varies at the level of individual plants as well as between populations and species. This variation can be in terms of composition (the types of chemicals present) and diversity (the number and evenness of chemicals present). Plants require the right phytochemicals to appropriately respond to local biotic and abiotic conditions, as investment into secondary metabolism is costly (Kessler and Kalske 2018), and mismatches may impair plant survival (Massad et al. 2011). Understanding phytochemical responses to biotic and abiotic conditions is therefore vital for predicting plant community responses to scenarios of global change (Zhou et al. 2024).

**Figure 1:**
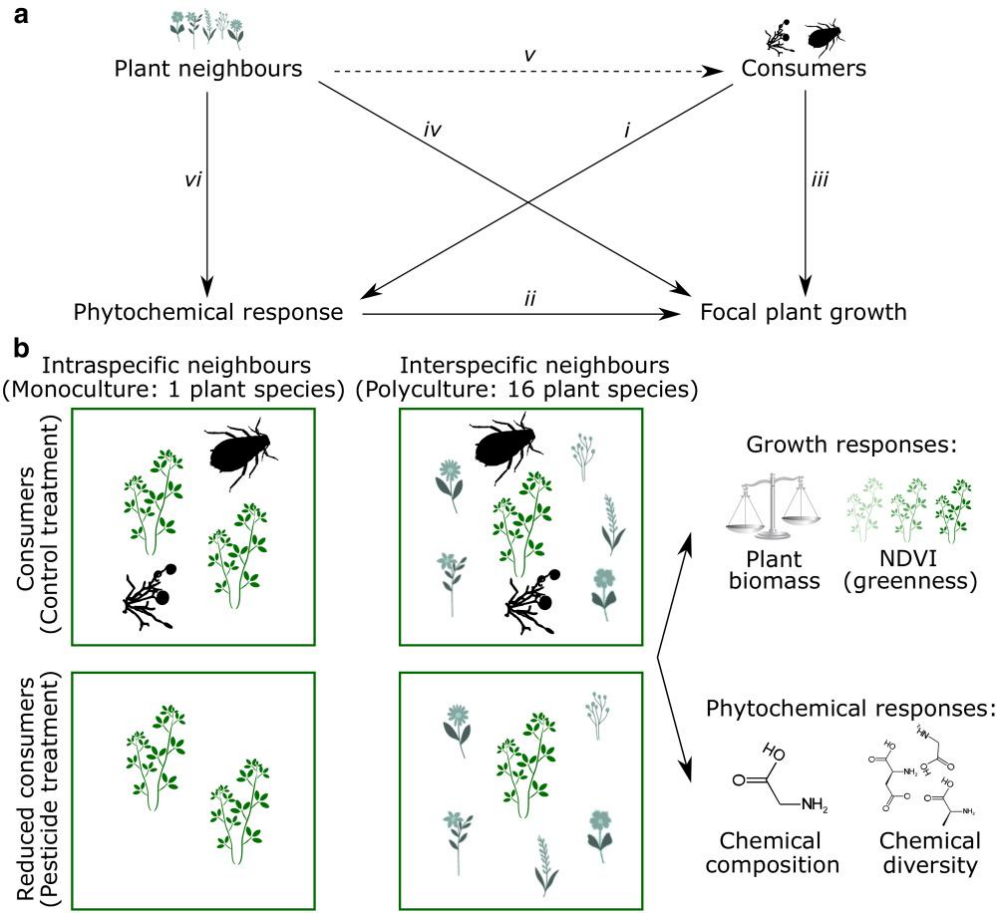
Summary of conceptual framework and experimental design. (a) Multiple drivers and their interactions affect focal plant growth and potentially phytochemical investment. (*i*) Attack from consumers like insect herbivores and fungal pathogens can stimulate induced defences, (*ii*) which plants use to maintain growth. (*iii*) Some plants invest mainly in constitutive defences, which mediate consumer impacts but do not change upon attack. (*iv*) Interspecific plant neighbours also affect focal plant growth directly or (*v*) indirectly through consumers. (*vi*) Neighbours could also directly stimulate inductive phytochemical changes. The holistic phytochemical response of plants involves within-plant trade-offs in response to these competing and potentially interacting pressures. Dotted line represents indirect effect. (b) Experimental design used in this study with the biomass and phytochemical response variables that we examined. We had plots for two focal species: *Lespedeza capitata* (legume) and *Andropogon gerardi* (C4 grass).

Multiple drivers may affect phytochemical responses, but previous work has overwhelmingly focused on insect herbivores (e.g. Fig. 1a  *i–iii*; Fraenkel 1959, Endara et al. 2023). Phytochemical diversity was originally theorized to be driven by coevolution with insects (Ehrlich and Raven 1964, Futuyma and Agrawal 2009). This viewpoint continues to dominate today, with every major hypothesis for phytochemical diversity involving consumers in some way (Wetzel and Whitehead 2020). Despite this theoretical focus on consumers, competition (e.g. allelopathy; Rice 1974, Ooka and Owens 2018, Fig. 1a  *vi*) and resource availability also affect phytochemical concentrations and composition, both ecologically (Hemming and Lindroth 1999, Hahn et al. 2021) and evolutionarily (Bryant et al. 1983, Coley et al. 1985, Zandt 2007). To date, there have been few attempts to consider the interacting effects of consumers, competitors and resource availability on phytochemical composition and diversity, with synthetic reviews typically focusing on only one driver (e.g. Latif et al. 2017, VanWallendael et al. 2019, Endara et al. 2023). This trend in the literature poorly reflects reality, where plants must simultaneously respond to multiple stressors and balance their phytochemical responses among them (Cipollini 2004, Fig. 1a). Individuals may exhibit within-plant trade-offs, where investment to withstand one stressor necessitates lower investment into other chemicals responding to other stressors, either by changing the concentrations of given chemicals or by changing the chemicals produced. Therefore, understanding overall phytochemical composition and resource investment in plants requires inspecting multiple factors simultaneously (Cipollini 2004, Agrawal 2007).

The interaction between plant neighbours and consumer pressure could be particularly influential. Interspecific neighbours can have direct effects on plant growth through competition for resources (Fig. 1a  *iv*), but also indirect effects through altering consumer communities (Fig. 1a  *v*; Turner and Schweitzer 2024). These interactions can strongly affect plant biomass, as revealed in field experiments where plant responses to herbivory (e.g. aboveground biomass, normalized difference vegetation index [NDVI]) varied with plant neighbourhood (Seabloom et al. 2018, Zaret et al. 2022). However, these studies did not examine phytochemistry, so it remains unclear if plant biomass changes caused by competition–consumer interactions were also seen in phytochemical responses. For example, does phytochemistry indirectly vary with interspecific neighbours via the effect different plant species can have on consumers (Fig. 1a  *v* → *i*), or do focal plants respond chemically to interspecific neighbours (Fig. 1a  *vi*), or both? If induced changes are evident, do plants change the types of compounds produced (phytochemical composition), or the number of compounds produced (phytochemical diversity)? To our knowledge, whether the effects of interactions between plant competitors and consumers involve phytochemical changes has not been explored under field conditions.

Phytochemical composition varies among and within species, but theory suggests that different species should also *respond* in different ways to the same environment (Agrawal 2007). For example, the carbon-nutrient balance hypothesis predicts that species with faster growth rates are better able to alter phytochemical investment by using induced rather than constitutive defences (Bryant et al. 1983). As variation among species influences both ecological and evolutionary interactions (Kessler and Kalske 2018, Glassmire et al. 2023), we need to understand whether different species in the same community show different abilities to phytochemically respond to interacting stressors. However, little work has examined intraspecific chemical responses of co-occurring species under field conditions. Buzhdygan et al. (2025) recently found that legumes and grasses were the two key functional groups driving energy dynamics in their study grassland, with legumes increasing herbivory among plant species in the community and grasses reducing it. Grasses and legumes also show high variability in nutrient ratios both within and between species (Borer et al. 2015), which could affect the type and quantity of phytochemicals they produce (Endara et al. 2023). Further, consumers and competitors interactively alter nutrient ratios of both grasses and legumes (Borer et al. 2015). This evidence suggests that grasses and legumes may show diverging phytochemical responses to consumers and interspecific neighbours with potential consequences for ecosystem dynamics. Legumes and grasses thus provide good models for testing if species’ responses differ. Here, we explore the possibility of species responding differently to biotic interactions using two grassland species: *Andropogon gerardi* (C4 grass) and *Lespedeza capitata* (legume).

We use a 15-year-old grassland experiment (Fig. 1b), manipulating intra- and interspecific neighbours (monocultures vs. 16-species polycultures) and reducing consumers (i.e. arthropods, foliar fungi, and soil fungi) to ask:

1. Do plant biomass and phytochemistry both change with consumer reduction and neighbour identity, or are biomass and phytochemical responses decoupled? (Are relationships between treatments and plant biomass mediated directly through pathways *iii* and *iv*, or phytochemically through pathways *i* and *vi*)?
2. Do *A. gerardi* (C4 grass) and *L. capitata* (legume) show consistent phytochemical responses to consumer pressure and plant neighbours? (Are the supported relationships in Figure 1a consistent between species from different functional groups)?

Species vary in their stoichiometry and aboveground response to consumer manipulation (Borer et al. 2015, Seabloom et al. 2018), but the extent to which this is reflected in their phytochemistry remains unclear. Our study thus provides insight into how consumers and neighbours affect the phytochemistry of species under real-world conditions. We find pronounced interspecific variability in both underlying phytochemical composition and in responses to biotic change.

## Materials and methods

### Experimental design and sampling

The experiment is based within a large biodiversity experiment established in 1994 at Cedar Creek Ecosystem Science Reserve, Minnesota, USA (Fig. 1b). Cedar Creek has nitrogen-limited sandy soils, annual precipitation of ∼780 mm, and the mean annual temperature of 6.72°C (averaged from 1987 to 2016 at Cedar Creek weather station). Replicate 9 m × 9 m grassland plots were planted with between 1 and 16 plant species, from a pool of 17 possible species of forbs, legumes, C3 grasses, and C4 grasses. Regular weeding maintained planted diversity levels. Fences around the wider experiment excluded large mammalian herbivores. In 2008, experimental consumer manipulation subplots (1.5 m × 2 m) were established within these plots, reducing foliar fungi, soil fungi, and insect herbivores through the fortnightly application of pesticides. See Tilman et al. (2006) and Borer et al. (2015) for full experimental details.

We sampled *A. gerardi* and *L. capitata* leaves in the experimental plots from plants with either ‘intraspecific neighbours’ (monoculture: species occurring in single-species plots) or predominantly ‘interspecific neighbours’ (polyculture: species occurring in 16-species plots). For each neighbourhood level, we sampled two levels of consumer manipulation: ‘consumers’ (no consumer manipulation) and ‘reduced consumers’ (all consumers reduced through the application of soil fungicide, foliar fungicide and foliar insecticide; Fig. 1b).

On 1 August 2023, in the early afternoon, we took three replicate leaf samples per treatment for each species (one replicate from each of three plots per polyculture, and three replicates from one monoculture plot, as there was only one monoculture per species), with samples taken 15 years after beginning the pesticide treatment. We sampled fully developed leaves that did not show signs of fungal or insect damage from the length of the plant (Massad et al. 2017, Forister et al. 2020), as induced defences are expressed in undamaged leaves as well as damaged leaves (Zandt 2007, Farmer 2014), and this strategy reduces variation in metabolite induction that may be caused by different levels of damage on damaged leaves (Moreira et al. 2025). Within these criteria, leaves were haphazardly sampled. Each sample for *A. gerardi* consisted of 4–5 leaves taken from a single plant, while each sample for *L. capitata* consisted of 10–12 leaves taken from a single plant. In several cases, individual *L. capitata* plants were not large enough to provide the required 50 mg of dried leaf material for phytochemical analysis, and so leaves of comparable ages were taken from two nearby plants; there were no differences observed in the results between samples that used one or two plants. In total, we took 24 samples (2 species × 2 neighbour identity treatments × 2 consumer treatments × 3 replicate plants).

Sampled leaves were dried at 60°C for 72 h, hand-ground to a fine powder using a pestle and mortar, and stored at room temperature until phytochemical extraction. This storage procedure yields a comparable richness and total quantity of metabolites as freeze–drying samples (Khoo et al. 2015, Zolkeflee et al. 2021), enabling fine-scale characterization of phytochemical composition and abundance (Richards et al. 2015). Yields of lipid-soluble compounds may be reduced by oven drying (Khoo et al. 2015, Ling et al. 2015); we compensated for this by using multiple solvents to explicitly target lipid-soluble as well as aqueous-soluble compounds (see below).

### Leaf damage and growth response to treatments

We assessed damage by foliar fungi and insect herbivores on our sampled plants by visually inspecting an additional five haphazardly selected leaves (approximately 30% of leaves for both species). Tissue removal (chewing damage) from the margin or interior of leaves as well as leaf miner tracks were counted as insect damage, while lesions, rust spots, and mildew were counted as fungal damage. We used the training programme and techniques described in Xirocostas et al. (2022) to ensure accuracy. Damage was calculated as a percentage of leaf removed/diseased, rather than absolute area, to account for different sizes of plants.

To test treatment effects on growth, aboveground biomass in each plot (from all species) was collected from a 1 m × 0.1 m strip using handheld clippers. Biomass was collected on 1 August after leaf sampling, sorted to species level, dried to constant mass, and weighed to the nearest 0.001 g. We also calculated the NDVI based on measurements of reflected radiation (reflectance) measured using an MSR5 multispectral radiometer (Cropspan, Inc.) on 13 September 2023 in the central 1.5 m^2^ area of each plot (avoiding clipped area). NDVI was calculated using the red (660 mm): near-infrared (830 mm) reflectance ratio and provides an estimate of cover that integrates over the whole plot, by broadly measuring ‘greenness’ (Gu et al. 2007). It therefore provides an alternate measure of plant biomass that is less vulnerable to potential randomness than a small strip and has been previously used at this scale to represent productivity (Zaret et al. 2022). However, the contribution of individual species to NDVI cannot be determined. To confirm the effect of our treatments on plant biomass, we took biomass and NDVI measurements for all experimental plots. This corresponded to nine 16-species plots and seventeen monoculture plots (one per species, as monoculture plots were not replicated at the species level).

### Phytochemical extraction

Our goal was to understand broad changes in phytochemical composition and diversity, so we took an untargeted ^1^H-NMR approach to examine holistic changes in metabolite production, rather than targeting specific compounds. This approach accounts for potential within-plant phytochemical trade-offs; an approach that only studies one chemical compound or class may find that it goes up or down, but total phytochemical investment may remain unchanged (Agrawal 2007). To capture as many phytochemicals as possible, we used multiple solvents to extract both aqueous-soluble and lipid-soluble phytochemicals. Although this wide-spectrum technique is uncommon, it builds a more complete picture of plant phytochemistry than more common approaches (Verpoorte et al. 2007).

We weighed 50 mg of each ground leaf sample and added 1.6 ml of deuterated solvents (0.4 ml CD_3_OD; 0.4 ml phosphate buffer in D_2_O containing 0.1% of internal standard trimethylsilylpropanoic acid; 0.8 ml CDCl_3_). These solvents extracted both lipid-soluble and aqueous-soluble chemicals into separate fractions. The lipid-soluble fraction captured lipids and terpenoids, while the aqueous-soluble fraction captured phenolics, flavonoids, amino acids, sugars, and most other classes of secondary metabolite, including some terpenoids (Verpoorte et al. 2007). We had 48 extracts in total (24 samples × 2 phytochemical fractions per sample), which were analysed using ^1^H-NMR spectroscopy. Raw ^1^H-NMR spectra were binned into 0.05 ppm increments from 0.5 to 9.5 ppm (aqueous fraction; 170 bins total) and from 3.3 to 11.3 ppm (lipid fraction; 159 bins total), with values representing the integral (area under the curve) in each bin. Data were pareto-scaled and normalized to a randomly chosen sample in preparation for analysis using MetaboAnalyst 5.0, following the protocol in Pang et al. (2021). For full details of phytochemical extraction and spectral acquisition, see Supplementary Methods S1: Phytochemical extraction.

### Statistical analysis

All statistical analyses were carried out in R v4.1.1 (R Core Team 2021). See Supplementary Code S1. Note that for both species, the monoculture replicates are drawn from one plot, as there was only one monoculture plot per species.

#### Leaf damage and growth responses to treatments

We tested for the effect of consumer manipulation, plant neighbours, and their interaction on total plant damage using two-way ANOVA, as well as in separate analyses for fungal damage and insect damage. Damage rates were transformed (log_10_[damage + 1]) to meet assumptions.

To generally explore how treatments affected NDVI and aboveground biomass, we tested for the effect of consumers, neighbour identity, and their interaction using two-way analysis of variance (ANOVA), for all plots in the wider experiment. We also compared the specific values of NDVI for *A. gerardi* and *L. capitata* monoculture plots between consumer treatments and compared the biomass of *A. gerardi* and *L. capitata* between consumer versus reduced consumer and intraspecific versus interspecific neighbourhood treatments. We did not carry out formal statistical analysis for our focal species comparisons, as there is only one monoculture plot per species, and we only looked at monoculture plots for NDVI as the contribution of individual species to NDVI cannot be determined in polyculture. For the biomass response, we compared the total biomass of our focal species (i.e. not standardized to the per-plant level) to capture population-level patterns.

#### Phytochemical differences between species

All the following analyses were performed separately for the aqueous fraction and the lipid fraction. To our knowledge, the phytochemistry of these two species has never been examined using ^1^H-NMR spectroscopy. As a result, no reference databases exist, which limits unambiguous phytochemical identification and quantification (Yesiltepe et al. 2022). Our approach therefore looked for holistic changes in overall phytochemical composition and diversity rather than quantifying specific compounds. Nevertheless, our comparisons of both composition and diversity used peak area (directly proportional to concentration in ^1^H-NMR spectroscopy), and so our analysis incorporated quantitative changes in phytochemistry. While our sample size was small (three replicates per treatment per species, with the three monoculture samples drawn from one plot), our main conclusions were consistent for multiple analytical choices (see Supplementary Methods S2: Robustness of results).

We first explored phytochemical composition differences (i.e. the types of chemicals present) between *A. gerardi* and *L. capitata*. While underlying species differences in composition are not the specific target of our study questions, they provide important context for interpreting the results. We performed a principal components analysis on the binned spectral data and visualized the first two principal components (aqueous fraction, 83.1% of total variation explained; lipid fraction, 81.5%). To test whether the centroids of the two species in multivariate space were equivalent, we used permutational multivariate analysis of variance (PERMANOVA) using the ‘adonis2’ function and Euclidean distances in the package vegan v.2.6-4 (Oksanen et al. 2022). To test whether total intraspecific variation differed between the two species, we compared the dispersion between the two species using the ‘betadisper’ function in vegan. To confirm which principal components contributed to differences between the two species, we also tested for differences in PC1 and PC2 values between the two species using one-way ANOVA.

We next explored differences in phytochemical diversity (i.e. the total number and evenness of chemicals present) between species. We calculated phytochemical diversity (1 − *D*) following Richards et al. (2015). Briefly, we normalized the binned data, so each sample had a total area of 100, and calculated *D* as *D* = Σ(*n*/*N*)^2^, where *n* is the integral (area under the curve) in a specific bin, and *N* is the number of bins (*N* = 170, aqueous fraction; *N* = 159, lipid fraction). We tested for differences in phytochemical diversity between the two species using one-way ANOVA.

Finally, we investigated the types of chemicals that lead to phytochemical differences between species. We identified ‘modules’ (sets of shared chemical bins that covary across samples) using a weighted network approach (Richards et al. 2018). Chemical bins that are more strongly correlated across samples are more closely related in the network and may indicate a certain class of phytochemical that appears across multiple samples. These closely related chemical bins were organized into modules (minimum module size = 3 bins) using the ‘blockwiseModules’ function in the WGCNA package (Langfelder and Horvath 2008, 2012) using a soft threshold to separate meaningful peaks from baseline noise. See Richards et al. (2018) and Supplementary Code S1 for full details. This procedure yielded seven modules of chemical bins. We plotted the loading of each chemical bin against PC1 and PC2 from the principal components analysis to visualize which modules contributed to higher or lower scores of PC1 and PC2 (Supplementary Figure S1). To identify the types of chemicals described by each module, we assembled a large database of previously published ^1^H-NMR chemical bins for a range of compounds from grasses and forbs (Supplementary Table S1). We compared the chemical bins of our modules to these published bins and to Table S6 of Richards et al. (2018) to identify the types of chemicals driving differences between our species. Identities of compounds with particularly strong correspondences were then confirmed using MestReNova (Mestrelab Research). This analysis was only carried out for the aqueous fraction, given the lack of previously published lipid chemical data (see Supplementary Table S1).

#### Phytochemical responses to neighbour identity and consumers

We repeated all the above analyses for both species, testing the interactive effects of consumer pressure and neighbour identity. We separated the data by species and tested the effect of consumers (control vs. pesticide) and intra- vs. interspecific neighbours (monoculture vs. polyculture) for each species separately, with three plants per treatment combination. We did not test these effects using the combined dataset of both species, as the two species were highly divergent (see Results) and so any signal would be dominated by this interspecific difference. In other words, rather than comparing *A. gerardi* versus *L. capitata* as above, we compared intraspecific vs. interspecific neighbours and consumers versus reduced consumers and their interactions for each species separately for all the above analyses.

For these treatment comparisons within species, we carried out two extra analyses. After identifying modules of chemical bins (10 modules for *A. gerardi*, 12 modules for *L. capitata*) and plotting these modules against PC loadings for each species (Supplementary Figures S2 and S3), we extracted module eigenvalues for each sample, which represent the relative occurrence of that module in the sample. We tested whether modules were affected by consumer or neighbour treatments and their interaction using two-way ANOVA. Second, we tested whether closely related samples in their aqueous-soluble phytochemical composition were also more closely related in their lipid-soluble phytochemical composition. For each species, we constructed a dendrogram of samples for the aqueous fraction and a dendrogram of samples for the lipid fraction (Supplementary Figures S4 and S5), using the ‘hclust’ function (Ward’s clustering criterion on Euclidean distances) in the dendextend package (Galili 2015). We compared the similarity between the two dendrograms using Baker’s Gamma (Baker 1974), which ranges from −1 (the dendrograms are much more dissimilar than expected by chance) to 1 (the dendrograms are much more similar than expected by chance). This test was implemented using the ‘cor_bakers_gamma’ function in dendextend. To test the significance of these comparisons, we generated 1000 random rearrangements of our dendrograms and repeated the test for each rearrangement. We calculated the permuted *P*-value as the proportion of random rearrangements that yielded a Baker’s Gamma larger than the observed value (Supplementary Figure S6).

## Results

### Leaf damage and growth responses to treatments

Pesticide application successfully reduced damage. Total leaf damage from consumers was 4.5% in control plots and 1.1% in pesticide plots (Supplementary Figure S7a). Consumer treatment interacted with neighbour treatment (*F*_1,20_ = 6.89, *P* = .016): reduction of consumers led to a greater damage decrease in the monocultures than the polycultures, because overall damage was lower when plants were with interspecific as opposed to intraspecific neighbours. This trend was largely driven by fungal damage (Supplementary Figure S7c), as insect damage was very low in 2023 (average of 0.78%) and did not significantly vary with either treatment (Supplementary Figure S7b).

**Figure 2:**
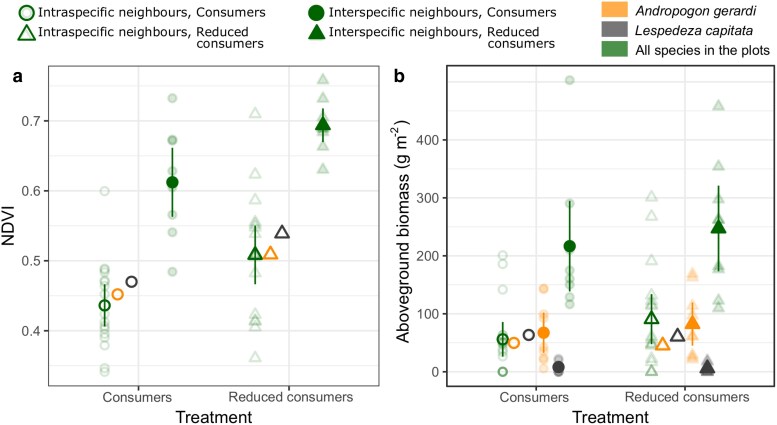
Effect of neighbour identity and reduced consumers on (a) NDVI and (b) total aboveground biomass. Darker points and error bars are mean ± 95% CI; lighter transparent points are individual experimental plots. For combined values of all species in the experiment, *N* = 17 monoculture plots and *N* = 9 16-species plots per consumer treatment. For *A. gerardi* and *L. capitata*, *N* = 1 monoculture plot and *N* = 9 16-species plots per consumer treatment. Note that no value for NDVI can be assigned to *A. gerardi* or *L. capitata* in 16-species plots.

Neighbour identity and consumer reduction had additive effects on plant NDVI and biomass (Fig. 1a  *iii*, *iv*). Across the whole experiment (all 17 species), NDVI in reduced-consumer plots was 1.14 times higher than plots with consumers (*F*_1,48_ = 14.45, *P* < .001) and was 1.37 times higher in the 16-species plots than in monoculture plots (*F*_1,48_ = 75.10, *P* < .001; Fig. 2a). This was also observed for the focal species: NDVI was 1.13 and 1.14 times higher in reduced-consumer monocultures relative to plots with consumers for *A. gerardi* and *L. capitata*, respectively (Fig. 2a). Aboveground biomass followed a similar trend as NDVI. Total biomass in plots where plants had interspecific neighbours was 3.1 times higher than monoculture plots (*F*_1,48_ = 34.52, *P* < .001) and was 1.2 times higher in reduced-consumer plots than consumer plots, though this was not significant (*F*_1,48_ = 1.67, *P* = .202; Fig. 2b). However, neighbour identity had varying relationships with the biomass of the two focal species. *Andropogon gerardi* biomass was up to 1.8 times higher when occurring with interspecific neighbours than with intraspecific neighbours, a trend consistent across consumer treatments (Fig. 2b), suggesting facilitation rather than competition. In contrast, *L. capitata* biomass was up to 10.4 times higher when with intraspecific neighbours only (Fig. 2b). Consumer effects were seemingly weak, with little effect of reducing consumers on the aboveground biomass of focal species (Fig. 2b).

### Phytochemical differences between species

**Figure 3:**
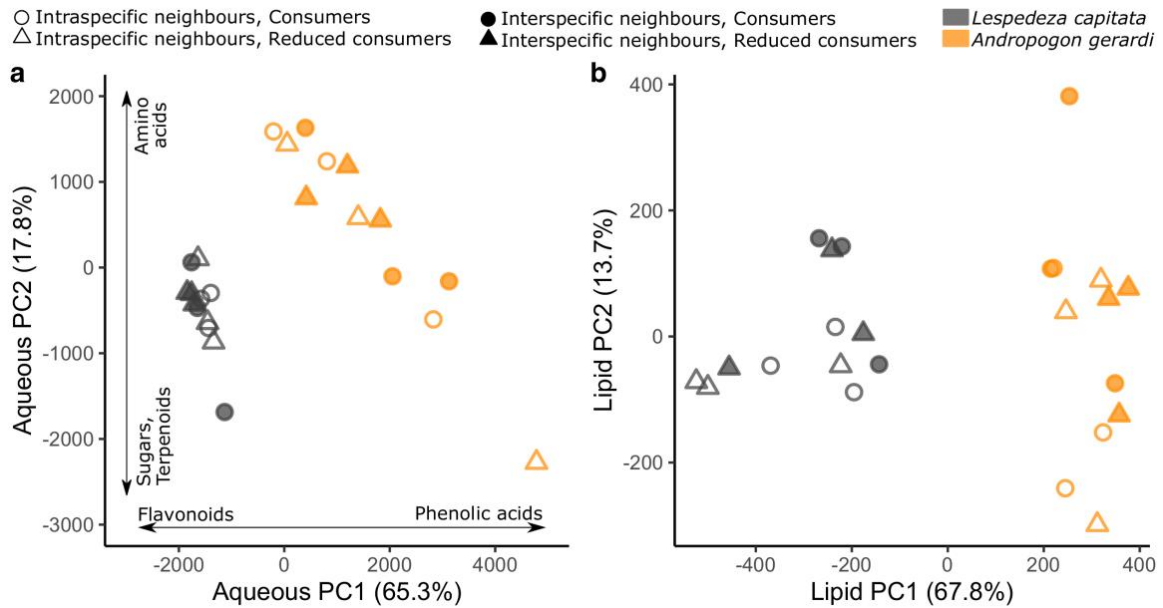
Phytochemical composition of *A. gerardi* and *L. capitata* expressed using principal components analysis for (a) aqueous fraction and (b) lipid fraction. For the aqueous fraction, broad chemical classes contributing to the PC axes are indicated. Each dot is a sample (*N* = 24 for aqueous fraction; *N* = 24 for lipid fraction).

**Figure 4:**
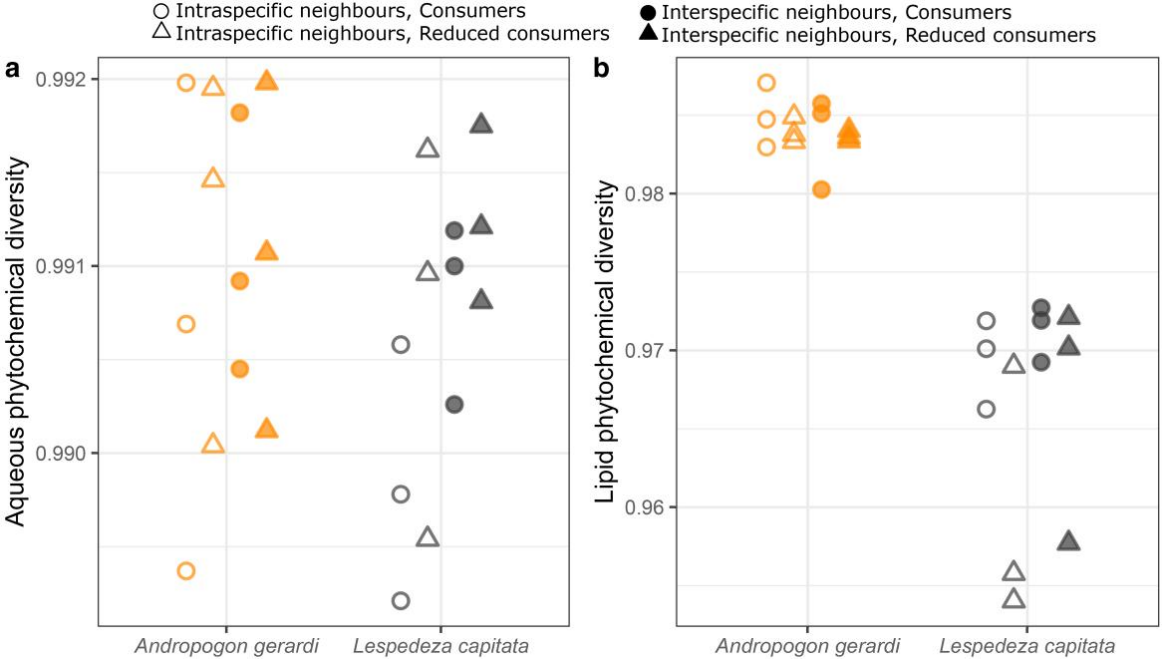
Phytochemical diversity for both the aqueous fraction (a) and the lipid fraction (b). Each dot is a sample (*N* = 24 for aqueous fraction; *N* = 24 for lipid fraction).

*Andropogon gerardi* and *L. capitata* differed in phytochemical composition (Fig. 3; Table 1). Both the aqueous and lipid fractions showed significant differences between the species (Table 1a), which indicates differential investment into (i) phenolic acids and flavonoids (PC1, Fig. 3a), and (ii) amino acids and sugars/terpenoids (PC2, Fig. 3a). These differences are supported by the significant PERMANOVA result for both extracts (Table 1a). *Andropogon gerardi* also showed higher intraspecific variation than *L. capitata* in the aqueous fraction (Fig. 3a; Table 1a). *Lespedeza capitata* showed higher quantities of flavonoids and terpenoids, while *A. gerardi* composition comprised more simple secondary metabolites such as phenolic and amino acids (Fig. 3a; Supplementary Figure S1; Supplementary Tables S1, S2a). Despite these compositional differences, the two species did not differ in overall aqueous phytochemical diversity (Fig. 4a; Table 1b). However, *A. gerardi* had significantly higher lipid phytochemical diversity (Fig. 4b; Table 1b).

**Table 1:** *P*-values for all statistical tests comparing phytochemical composition and diversity across species and treatments.

Metric	Fraction	Species	Intraspecific vs. interspecific neighbours		Consumers vs. reduced consumers	
		*Ag* vs. *Lc*	Within *Ag*	Within *Lc*	Within *Ag*	Within *Lc*
(a) Phytochemical composition						
PERMANOVA	Aqueous	**<**.**001**	.953	.**050**	.912	.781
	Lipid	**<**.**001**	.157	.072	.214	.107
Dispersion	Aqueous	.**015**	.414	.970	.838	.340
	Lipid	.227	.602	.752	.676	.619
PC1	Aqueous	**<**.**001**	.802	.179	.857	.879
	Lipid	**<**.**001**	.212	.142	.698	.116
PC2	Aqueous	.**011**	.713	.**019**	.512	.195
	Lipid	.939	.263	.182	.**035**	.469
(b) Phytochemical diversity						
1-D	Aqueous	.351	.799	.090	.645	.139
	Lipid	**<**.**001**	.469	.213	.653	.058

Ag = *Andropogon gerardi*; Lc = *Lespedeza capitata*. *P*-values <.05 are bolded. Values for species comparisons are from analyses combining both species; values for treatments are from analyses of each species separately. PERMANOVA tests the *H*_0_ that centroids of the groups are equivalent. Dispersion tests the *H*_0_ that the total multivariate variation is equal among groups. 1-D represents phytochemical diversity.

### Phytochemical differences between treatments within species

For both species, there was no similarity in clustering between the aqueous and lipid fractions (*A. gerardi*, Baker’s Gamma = 0.13, *P* = .098; *L. capitata*, Baker’s Gamma = −0.09, *P* = .590), suggesting the two fractions, and their responses to neighbours or consumers, are independent (Supplementary Figures S4–S6).

#### Lespedeza capitata

**Figure 5:**
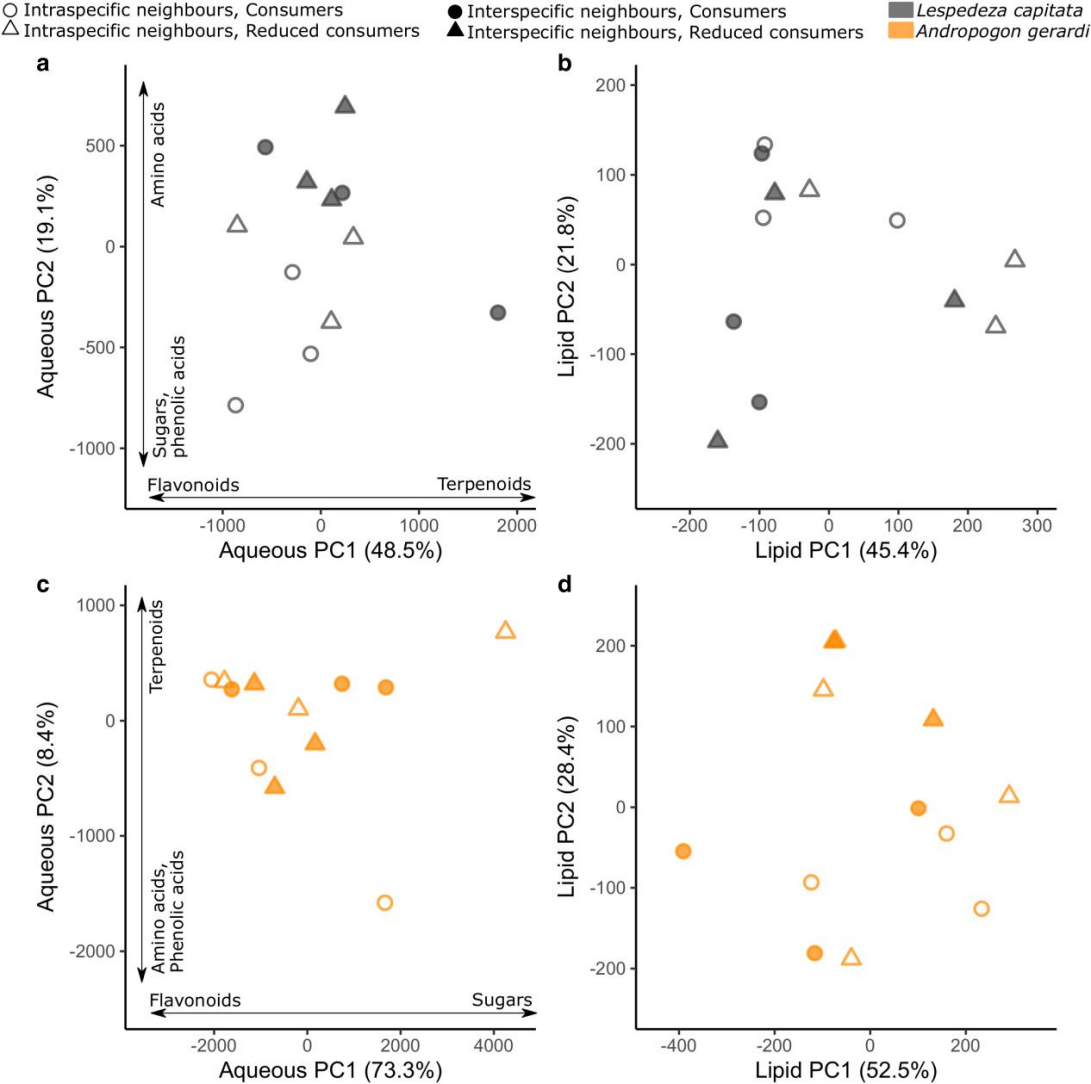
Phytochemical composition differences between samples in different treatments expressed using separate principal component analyses for *L. capitata* (a, b) and *A. gerardi* (c, d) for both the aqueous fraction (a, c) and the lipid fraction (b, d). Each dot is a sample (*N* = 12 for aqueous fraction and *N* = 12 for lipid fraction for each species). For the aqueous fraction, broad chemical classes contributing to the PC axes are indicated. Note that because principal components were calculated separately for each species, chemical classes load differently for the two species.

Phytochemical composition of the *L. capitata* aqueous fraction was affected more by neighbour identity than consumer reduction (Fig. 1a  *vi* supported, *i* unsupported), which was driven largely by higher values of PC2 with interspecific neighbours (Fig. 5a; Table 1a). Similarly, there was a marginally significant effect of neighbours in the *L. capitata* lipid fraction, where plants with interspecific neighbours had slightly lower PC1 values than those with intraspecific neighbours (Fig. 5b). These results were also seen in the clustering analysis, with evidence of clustering according to neighbour treatment but not consumer treatment (Supplementary Figure S5). There was also a marginal relationship between neighbour identity and aqueous phytochemical diversity where plants with interspecific neighbours had higher diversity than those with intraspecific neighbours (Fig. 4a; Table 1b), and a marginal relationship between consumer manipulation and lipid phytochemical diversity, where plants had higher diversity when consumers were present (Fig. 4b; Table 1b). There were no interactive effects of neighbour identity and consumer reduction on overall phytochemical composition or diversity.

**Figure 6:**
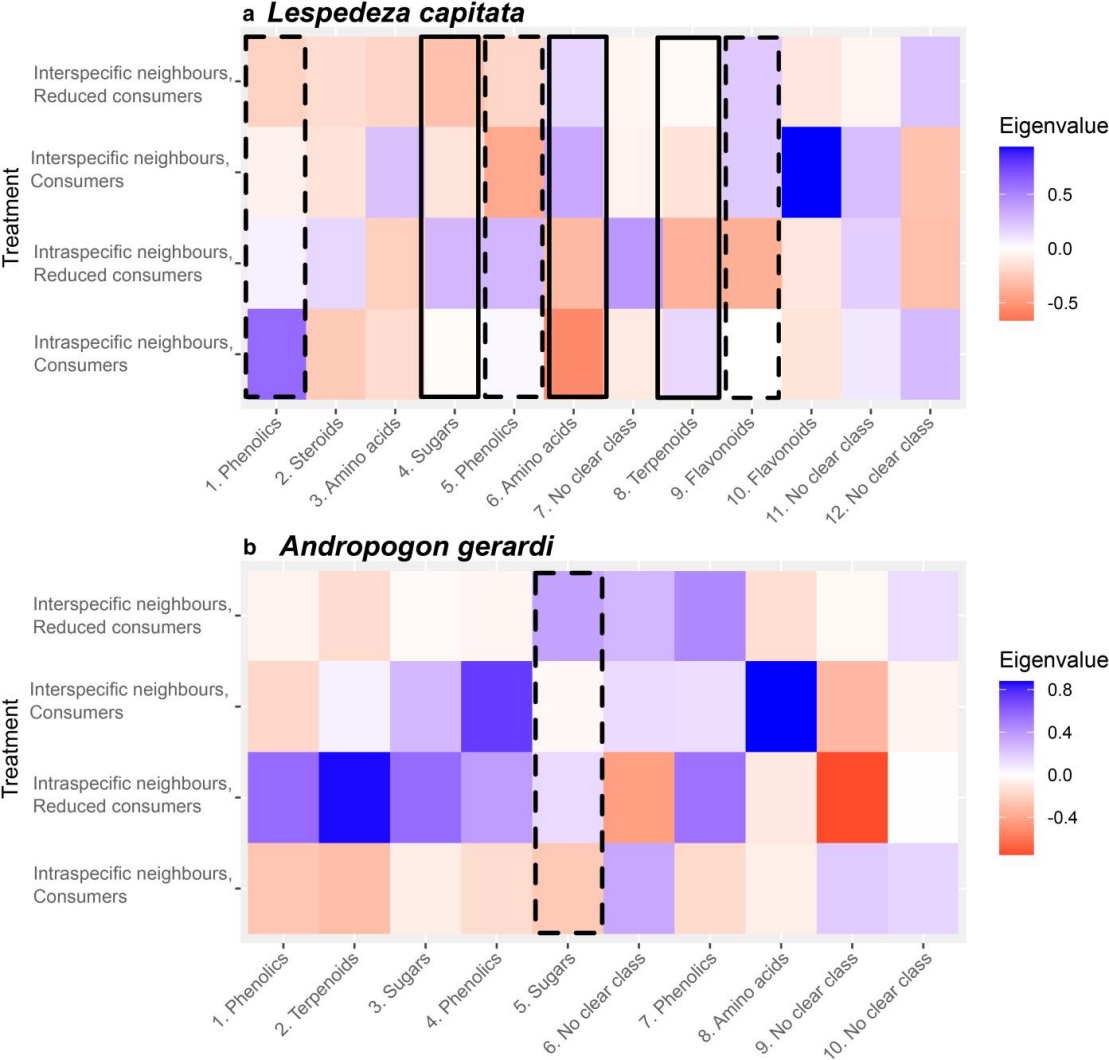
Modules of chemical bins for (a) *L. capitata* and (b) *A. gerardi*, with the chemical class comprising each module indicated (see Supplementary Table S2 for full details; some modules did not align with a clear phytochemical class). Panel heat represents the average eigenvalue across the three replicates of each treatment combination, with blue (higher eigenvalues) representing upregulation of the module in the treatment and red (lower eigenvalues) representing downregulation of that module in the treatment. Solid black outlines highlight modules that statistically differed between treatments, while dashed lines show non-significant patterns across treatments.

In sum, *L. capitata* phytochemistry was affected by neighbours but not consumers, except for a marginal relationship with consumers in lipid phytochemical diversity. To further investigate these changes, we examined the modules of chemical bins (Supplementary Figure S2; Supplementary Table S2b). Of the 12 modules identified for *L. capitata*, six showed directional trends of which three were statistically significant (Fig. 6a). Sugars (especially glucose and sucrose, module 4) were higher when occurring with intraspecific neighbours only *F*_1,9_ = 13.47, *P* = .005), while amino acids (module 6) were higher with interspecific neighbours (*F*_1,9_ = 11.03, *P* = .009). Consumers and neighbours also interacted to affect terpenoids (module 8, *F*_1,8_ = 6.68, *P* = .032): terpenoid concentrations were highest in plants grown with intraspecific neighbours and consumers, lowest in plants growing with intraspecific neighbours with reduced consumers, with intermediate values when growing with interspecific neighbours. Plants with intraspecific neighbours also tended to have higher investment in phenolics (modules 1, 5) and lower investment in flavonoids (module 9), though not significantly (Fig. 6a).

#### Andropogon gerardi

In contrast to *L. capitata*, *A. gerardi* showed negligible phytochemical responses to either consumer or neighbour treatments (Figs. 4, 5c; Supplementary Figure S4; Table 1; Fig. 1a  *i* and *vi* unsupported). The one exception was a trend for plants with reduced consumers to have higher PC2 values in lipid phytochemical composition (Fig. 5d; Table 1a). Analysis of chemical modules supported these results (Fig. 6b; Supplementary Figure S3; Supplementary Table S2c). Of the 10 modules identified, none showed a significant relationship with neighbour identity, while sugars (module 5) showed a non-significant increase in plants with reduced consumers (Fig. 6b).

## Discussion

In this study, we asked two questions: (1) Do plant biomass and phytochemistry both change with consumer reduction and neighbour identity, or are biomass and phytochemical responses decoupled?—Our findings suggest consumers and neighbours affect biomass and NDVI measures, but that these changes were not always reflected in phytochemistry. (2) Do *A. gerardi* and *L. capitata* show consistent phytochemical responses to neighbour and consumer manipulations?—We found that *A. gerardi* and *L. capitata* differed in both their underlying phytochemical composition and in their responses to consumers and neighbours. Collectively, our results suggest that neighbour identity can exert stronger effects on intraspecific phytochemical responses than consumer presence, but these potential effects differ between our two focal species.

### Distinct and species-specific effects of competitive environment and consumers on plant biomass and phytochemistry

Plant aboveground biomass and NDVI varied with consumer reduction and neighbour identity, but corresponding variation was not always seen in the phytochemistry of either *A. gerardi* or *L. capitata* (Question 1). *Andropogon gerardi* NDVI and biomass were higher when consumers were reduced (though the biomass results were non-significant) and when growing with interspecific vs intraspecific neighbours (Fig. 2), both of which reduced damage on plants. However, there was no change in *A. gerardi* phytochemistry (Fig. 5), aside from a small potential upregulation of sugars in plants with reduced consumers (Fig. 6). These results suggest that *A. gerardi* relies on more constitutive than induced defences (Waterman et al. 2021). Previous work has also found that *A. gerardi* shows little induced response to consumer pressure (Pittenger et al. 2020), suggesting the frequency or multiplicity of stressors faced by *A. gerardi* in this environment has favoured the development of constitutive phytochemical pathways. However, total intraspecific phytochemical variation of *A. gerardi* was higher than for *L. capitata* (Fig. 3). High intraspecific variation may itself reduce damage in a population as it provides a ‘moving target’ for herbivores (Massad et al. 2017). In sum, *A. gerardi* benefitted from consumer removal, at least in terms of NDVI. It also appeared highly competitive in 16-species communities—or may even be facilitated by other species—as indicated by its higher biomass when growing with interspecific neighbours (Fig. 2b). The stronger overall signal for NDVI versus biomass may be because NDVI averages productivity across a larger area of the plot, while biomass is estimated from a small strip that may be vulnerable to stochastic positioning of plants. There was no corresponding phytochemical response in *A. gerardi*, supporting pathways *iii* and *iv* (Fig. 1a).

*Lespedeza capitata* displayed different patterns to *A. gerardi* in both biomass and phytochemistry (Question 2). *Lespedeza capitata* average biomass was approximately 10 times lower when with interspecific versus intraspecific neighbours (Fig. 2b). This corresponded with phytochemical changes (Figs. 5, 6): sugars were higher when growing alone, while amino and phenolic acids were higher when grown with interspecific neighbours. Collectively, these results indicate that interspecific competition may impose an energetic cost on *L. capitata*. Sugars represent energy stores (Lastdrager et al. 2014), while amino acids and phenolics are derived through amino acid metabolism (Endara et al. 2023) and thus cheaper to produce for N-fixers like *L. capitata*. High amino acid levels are also indicative of stress (Witt et al. 2012, Hildebrandt et al. 2015). The growth and phytochemistry results collectively suggest that interspecific neighbours reduce resource availability or at least alter resource allocation for *L. capitata* which can be seen its chemical composition. These results were consistent regardless of consumer treatment, suggesting that interspecific plant neighbours exert a direct effect on phytochemistry (Fig. 1a  *vi*) rather than an indirect effect mediated by consumers (Fig. 1a  *v* → *i*).

Interspecific competitors also potentially affected *L. capitata*’s response to consumers. Without interspecific neighbours and when faced with consumers, *L. capitata* upregulated terpenoids (Fig. 6), which are effective but costly defence compounds (Wolf et al. 2011). When grown with interspecific neighbours, this response was lost, further suggesting interspecific competition reduced the energy stores of *L. capitata* and its ability to redirect defences appropriately. However, evidence for *L. capitata* phytochemical responses to consumer pressure is smaller than its phytochemical responses to neighbour identity (Table 1; Figs. 5, 6). This suggests *L. capitata* may also largely rely on constitutive defence, though we note an important caveat to this conclusion: as we only sampled once, we are less likely to detect induced defences unless consumption was very frequent. Our damage data suggests this condition is unlikely to be met, as total damage was low in 2023 (4.5% in control plots), which is lower than reported in other years at Cedar Creek (e.g. Borer et al. 2015, Brian et al. 2024). It may be that conditions were less suitable for consumers, especially insect herbivores (Supplementary Figure S7). This would limit the difference between control and pesticide treatments (Fig. 2b) and may underestimate the influence consumers can have on phytochemistry. Nevertheless, release from consumers clearly increased NDVI, both in our focal species and across the experiment generally (Fig. 2a), suggesting a significant effect of consumers on biomass.

Based on the above, we cautiously suggest that over local scales (defined here as 3 m^2^) and for the conditions of this study, interspecific competition exerts stronger effects than consumers on biomass (both species), and any induced phytochemical changes are due to neighbours (*L. capitata*). The greater responsiveness of *L. capitata* phytochemistry is predicted by theory: one prediction of the carbon-nutrient balance hypothesis states that species with faster growth rates are better able to alter phytochemical investment (Bryant et al. 1983). Trait data suggest *L. capitata* is the faster-growing species (Catford et al. 2019), which suggests its phytochemical profile may be more inducible in response to stressors. The current study adds to growing evidence that, within species, short-term phytochemical changes may be influenced by resources or plant neighbours more so than by consumers (Kigathi et al. 2019, Glassmire et al. 2023, Molleman et al. 2024), while acknowledging that underlying constitutive phytochemical composition may still be determined by long-term interactions between plants and consumers (Futuyma and Agrawal 2009). We recommend further work that investigates the varying abilities of different species to adapt phytochemically to the rapid consumer and community diversity changes predicted in the future.

### Differences between aqueous-soluble and lipid-soluble phytochemical investment

We found no congruence between sample clustering of the aqueous and lipid fractions, possibly indicating independent investment by plants into aqueous-soluble and lipid-soluble phytochemicals. We have paid little attention to lipid results in our discussion, partly because the lipid fraction was less responsive to treatment than the aqueous fraction, but also because there is very little published work on lipid-soluble phytochemicals (Supplementary Table S1). However, there were clear differences between the lipid diversity of the species (Fig. 4b) and possible responses of lipids to consumer pressure in both species (Table 1). Recent work has revealed the importance of lipid signalling in response to herbivory (Xing et al. 2024). Aqueous-soluble and lipid-soluble chemicals both support defence and stress responses but also energy storage and cell architecture and so drawing general implications from the different investment patterns between the two is difficult. Our results highlight an important gap in knowledge that could be filled if future work uses multiple solvents to gain a holistic view of plant phytochemical responses to change. These efforts will be enhanced by methods providing more conclusive identification of major chemical classes and their functional roles.

### Conclusion

Using a grassland field experiment, we found variation in the phytochemical composition and response to interspecific competition of two co-occurring plant species. How communities phytochemically respond to changes in biotic context may be contingent on the focal species in those communities, in a way that cannot necessarily be predicted from measures of plant biomass. We suggest that intraspecific phytochemical responses to different ecological conditions have not been investigated as rigorously as interspecific phytochemical diversity, and the direct effects of interspecific interactions require greater attention. Our results indicate that while constitutive phytochemistry may have developed in response to long-term interactions with consumers, the competitive context for a species may determine short-term induced phytochemical changes, though the low overall consumer impact in the study year provides an important caveat. The impacts of interspecific competition on phytochemical diversity (and the variation of impacts depending on focal species) revealed by our study provides impetus for more in-depth work into the interaction between plant diversity, phytochemical diversity and consumer diversity, and the directionality of these relationships under real-world conditions.

## Supplementary Material

plaf071_Supplementary_Data

## Data Availability

All data and code supporting the manuscript can be found in Zenodo: https://doi.org/10.5281/zenodo.17236625 (Brian 2025).
