# Supplementary material for: Plant neighbours, not consumers, drive intraspecific phytochemical changes of two grassland species in a field experiment
Source: AoB Plants. 2025 Dec 15;17(6):plaf071. doi: 10.1093/aobpla/plaf071 (PMC12721384; doi:10.1093/aobpla/plaf071)
Supplement: plaf071_Supplementary_Data [file plaf071_supplementary_data.pdf]

Plant neighbours, not consumers, drive intraspecific phytochemical changes of two grassland species in a field experiment

Supporting Information

Supporting Methods

S1: Phytochemical extraction 2

S2: Robustness of results 3

Supporting References 4

Supporting Figures

S1: Chemical modules across both species 5

S2: Chemical modules for *L. capitata* 6

S3: Chemical modules for *A. gerardi* 7

S4: Clustering analysis for *L. capitata* 8

S5: Clustering analysis for *A. gerardi* 9

S6: Baker's gamma distributions for both species 10

S7: Leaf damage results 11

Supporting Tables

S1: ^{1}H chemical shifts for plant metabolites 12

S2: Chemical shifts associated with all modules 19

Supporting Methods S1: Phytochemical extraction

Phytochemical extraction

We weighed 50 mg of each ground sample into an Eppendorf tube and added 1.6 mL of deuterated solvents (0.4 mL CD_3OD; 0.4 mL phosphate buffer in D_2O containing 0.1% (wt/wt) of internal standard TSP; 0.8 mL $CDCl_3$). We vortexed samples for 1 min, sonicated in a water bath at room temperature for 15 min then centrifuged at 14,700 g for 10 min. Following centrifugation, the sample tubes had three layers: the aqueous fraction on top, a solid layer of plant debris in the middle and the lipid fraction at the bottom. We removed the aqueous and lipid fractions with a pipette and placed them in separate tubes.

Spectra acquisition

Following separation, 600 µL of each fraction were transferred into 5 mm NMR tubes. All spectra were acquired on a Bruker Avance NEO 600 MHz equipped with a TCI Cryoprobe Prodigy (Bruker Biospin, Karlsruhe, Germany), operating at a proton frequency of 600.2 MHz at 298 K. For aqueous samples, ^1H spectra were acquired using the 1D version of the NOESYpr pulse sequence with water suppression by presaturation (noesygppr1D), with a spectral window of 20.8 ppm, a total spin-echo time of 10 ms, a relaxation time of 4 s, an acquisition time of 2.62 s and 64 scans. For lipid samples, the standard pulse-acquire pulse sequence (zg) was used, with a spectral window of 19.8 ppm, a relaxation time of 3 s, an acquisition time of 2.75 s and 64 scans. The TSP and TMS peak (0 ppm) were used as internal reference/standard for aqueous and lipid samples, respectively. After acquisition, the spectra were analysed in TopSpin (version 4.0.3; Bruker Biospin, Karlsruhe, Germany). A 0.3 Hz exponential line broadening function was applied before Fourier transformation for all spectra and automatic phase correction. Baselines were inspected and polynomial baseline correction of order 3 applied.

Raw ^1H-NMR spectra were then binned into 0.05 ppm increments from 0.5 to 9.5 ppm (aqueous fraction; 170 bins total) and from 3.3 to 11.3 ppm (lipid fraction; 159 bins total), with values representing the integral (area under the curve) in each bin range. Bins representing the residual solvent peaks of water (aqueous 4.7 – 4.9 ppm), methanol (aqueous

3.25 – 3.45 ppm) and chloroform (lipid 7.25 – 7.30 ppm) were removed, and data were then pareto-scaled and normalised to a randomly-chosen sample in preparation for analysis.

Supporting Methods S2: Robustness of results

Additional statistical tests

To test the robustness of our results, we carried out additional analyses using different subsets of the data. First, to remove potentially spurious variance, we excluded 50% of the chemical bins that had the lowest loadings on to PC1 and PC2 (i.e. we removed bins that did not contribute strongly to either PC). Second, we only analysed aqueous data with bins >5 ppm, as Richards et al. (2015) suggest that ecologically relevant phytochemicals occur largely above this value. Third, we treated each treatment combination as a single variable (i.e. 'monoculture control'; 'monoculture pesticide'; 'polyculture control'; 'polyculture pesticide'), rather than analysing monoculture vs. polyculture and control vs. pesticide in separate analyses. This enabled the detection of any interactive additive or dampening effects of the combined treatments that may not have been detected by analysing each set of treatments separately, though we did also test for consumer*neighbour interactions in our main analyses where possible (for the PERMANOVA and ANOVA tests). Fourth, we excluded one sample (*A. gerardi* sample 18) as a potential outlier, as it clustered very differently to all other *A. gerardi* aqueous samples (Fig. S4). Fifth, we tested whether PC3 from the principal components analyses was significantly related to treatments, to explore additional potentially relevant variation. Sixth, to complement the PERMANOVA and dispersion analyses, we also used the rank order of dissimilarities of binned chemical peaks (anosim function in vegan package). None of these analyses yielded qualitatively different results to the main analyses, and so we only present the main analyses in the main text. See Supplementary Code S1 for details of all additional analyses.

Influence of pesticide treatment on response variables

Based on our current and previous results, we think it is unlikely that pesticide treatments directly affected our phytochemical results. First, we searched the MestReNova database for the active ingredients of the pesticide compounds. None of these compounds were present in the sample spectra, suggesting that they did not directly influence the sample results.

However, there is the possibility of indirect effects, through (for example) providing a small fertilisation effect in the plots, which could change resource availability and hence phytochemistry. Evidence suggests that this is not the case. The class of fungicidal compounds used in this experiment (Triazoles) has been shown not to induce changes in the growth, height or leaf area of wild grasses (Paul et al. 1989), though less is known of their effects on non-agricultural legumes. Similarly, work examining the insecticides used in this study suggests minimal direct effects on plant growth (Brown et al. 1987) or foliar nitrogen (Syvertsen & Dunlop 2009). Finally, experimental work in greenhouses has shown that, in the absence of consumers, there are no difference between pesticide-treated and untreated plants, either above or below-ground (Seabloom et al. 2017). Collectively, these results suggest that our conclusions are not affected by any side-effect of direct pesticide influence on phytochemical composition or plant growth.

Supporting References

Brown, V.K., Leijn, M. & Stinson, C.S.A. (1987). The experimental manipulation of insect herbivore load by the use of an insecticide (malathion): the effect of application on plant growth. *Oecologia*, **72**, 377–381.

Paul, N.D., Ayres, P.G. & Wyness, L.E. (1989). On the use of fungicides for experimentation in natural vegetation. *Functional Ecology*, **3**, 759–769.

Richards, L. A., Dyer, L. A., Forister, M. L., Smilanich, A. M., Dodson, C. D., Leonard, M. D., & Jeffrey, C. S. (2015). Phytochemical diversity drives plant–insect community diversity. *Proceedings of the National Academy of Sciences*, **112**, 10973-10978.

Seabloom, E. W., Kinkel, L., Borer, E. T., Hautier, Y., Montgomery, R. A., & Tilman, D. (2017). Food webs obscure the strength of plant diversity effects on primary productivity. *Ecology Letters*, **20**, 505-512.

Syvertsen, J.P. & Dunlop, J.M. (2009). Imidacloprid has little effect on growth or drought tolerance of citrus rootstock seedlings without pests. *Proceedings of the Florida State Horticultural Society*, **122**, 81–84.

Supporting Figures

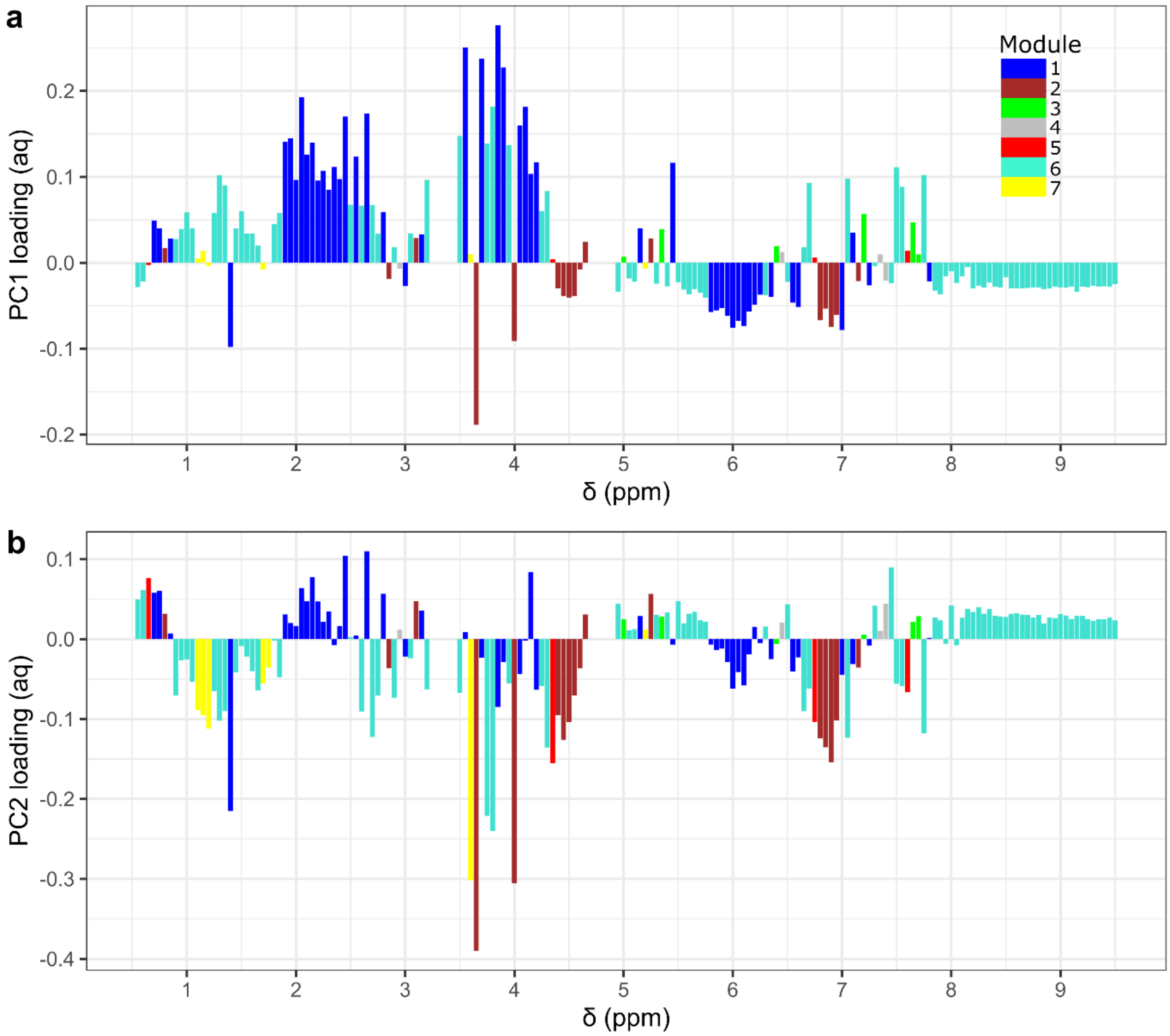

Fig. S1: Chemical modules identified from all 24 samples across both species (aqueous fraction) using weighted network analysis, plotted against their loadings from the overall principal components analysis. Strongly positive or negative values indicate contributions to high or low values of the principal component; values close to zero indicate minimal contribution to separation of samples along principal component axes. For module identity see Table S2a.

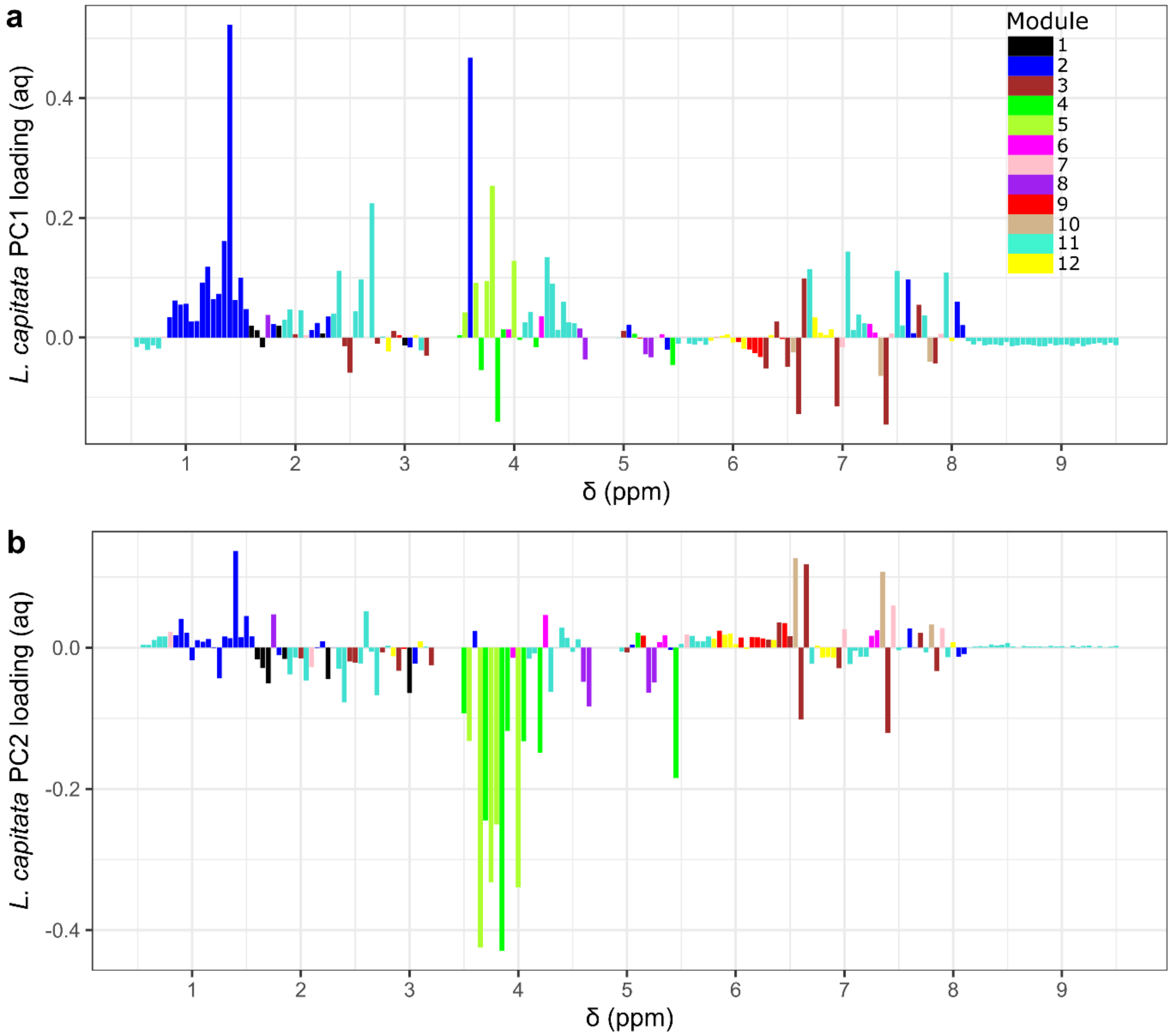

Fig. S2: Chemical modules identified from *L. capitata* (aqueous fraction) using weighted network analysis, plotted against their loadings from the overall principal components analysis. Strongly positive or negative values indicate contributions to high or low values of the principal component; values close to zero indicate minimal contribution to separation of samples along principal component axes. For module identity see Table S2b.

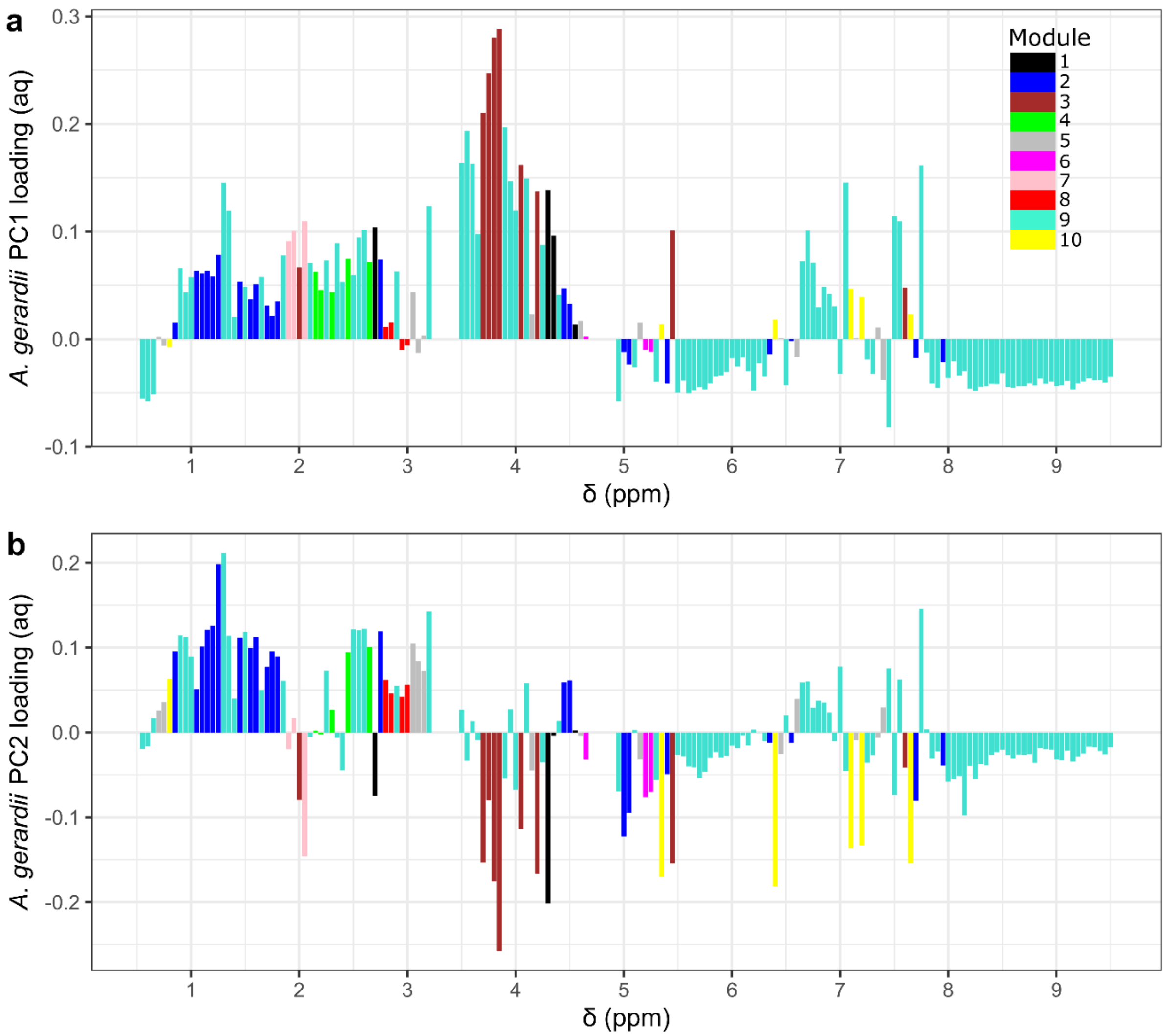

Fig. S3: Chemical modules identified from *A. gerardi* (aqueous fraction) using weighted network analysis, plotted against their loadings from the overall principal components analysis. Strongly positive or negative values indicate contributions to high or low values of the principal component; values close to zero indicate minimal contribution to separation of samples along principal component axes. For module identity see Table S2c.

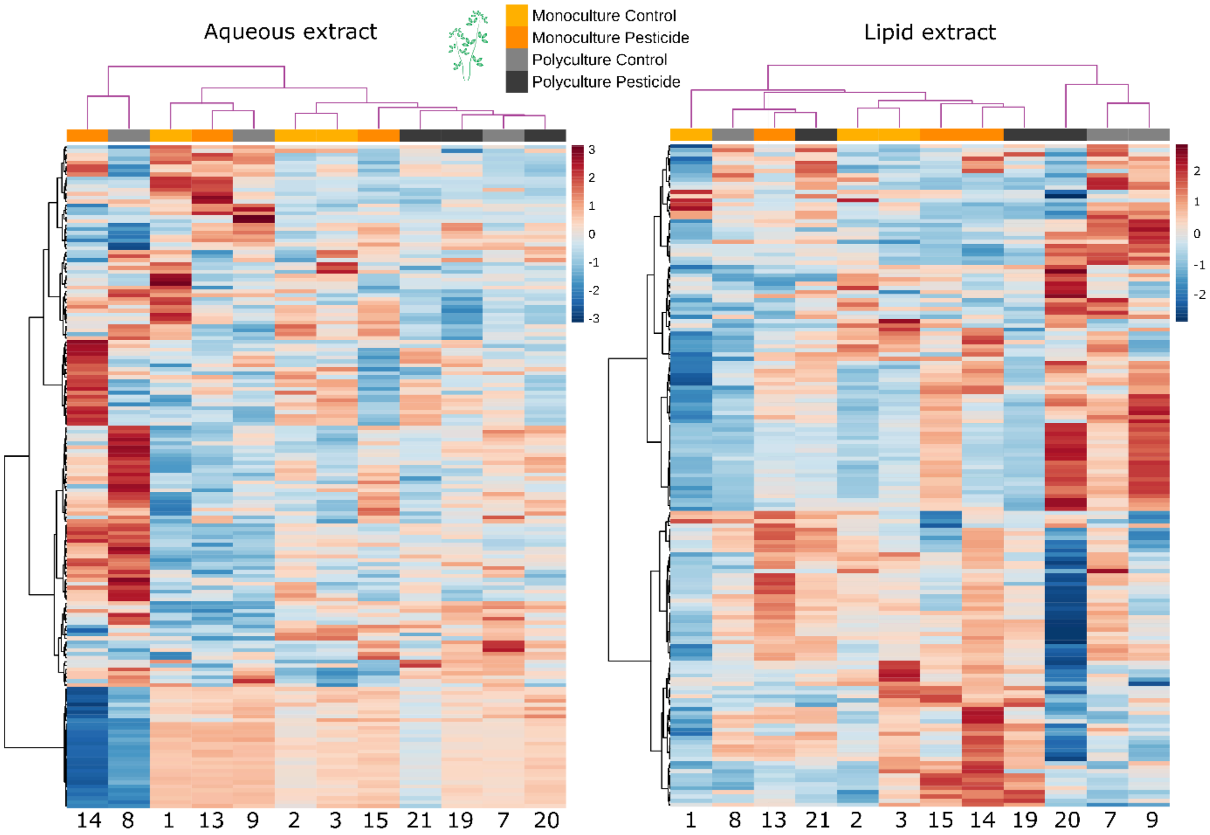

Figure S4: Results of clustering analysis for *L. capitata*. Rows are chemical shifts and columns are samples, with colour representing whether that chemical shift occurs at a high level (red) or low level (blue) in the sample. Numbers at the bottom of each column correspond to sample number and are consistent across two fractions (i.e. sample 14 is from the same plant for both fractions).

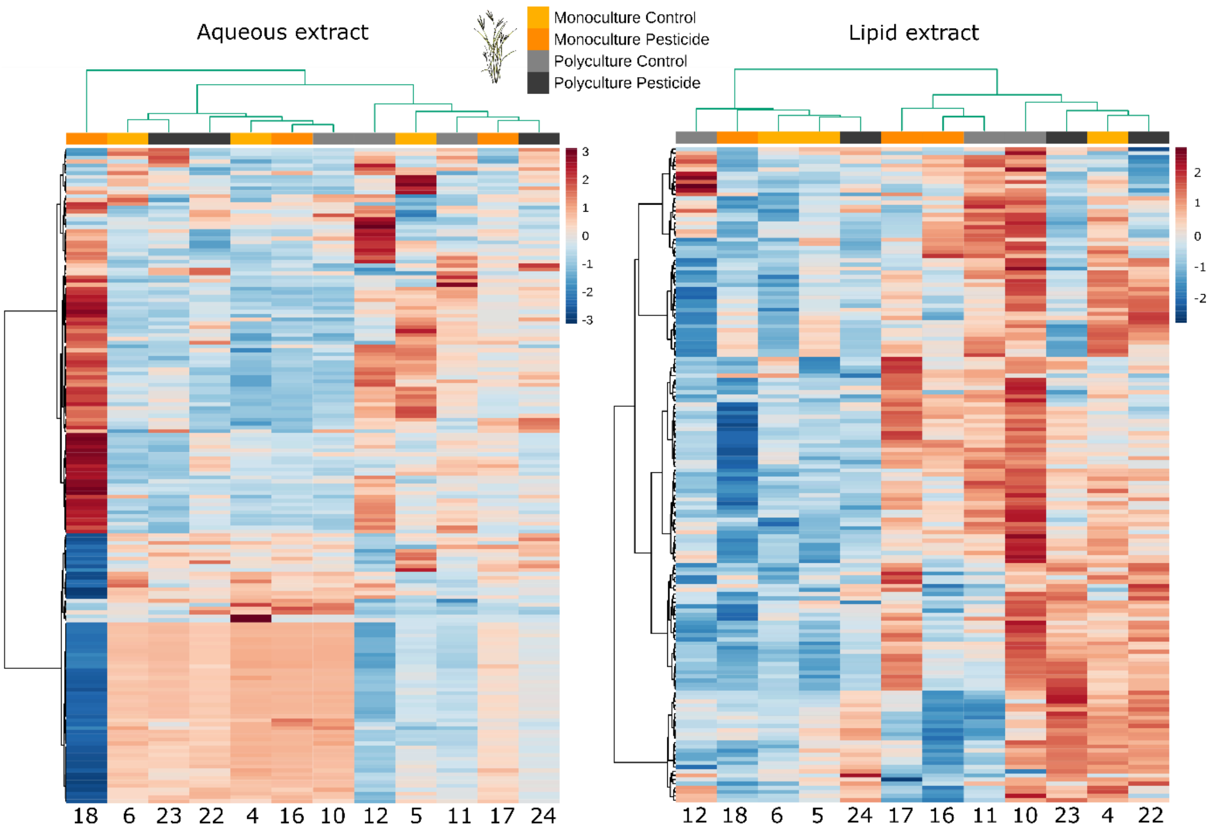

Figure S5: Results of clustering analysis for *A. gerardi*. Rows are chemical shifts and columns are samples, with colour representing whether that chemical shift occurs at a high level (red) or low level (blue) in the sample. Numbers at the bottom of each column correspond to sample number and are consistent across two fractions (i.e. sample 24 is from the same plant for both fractions).

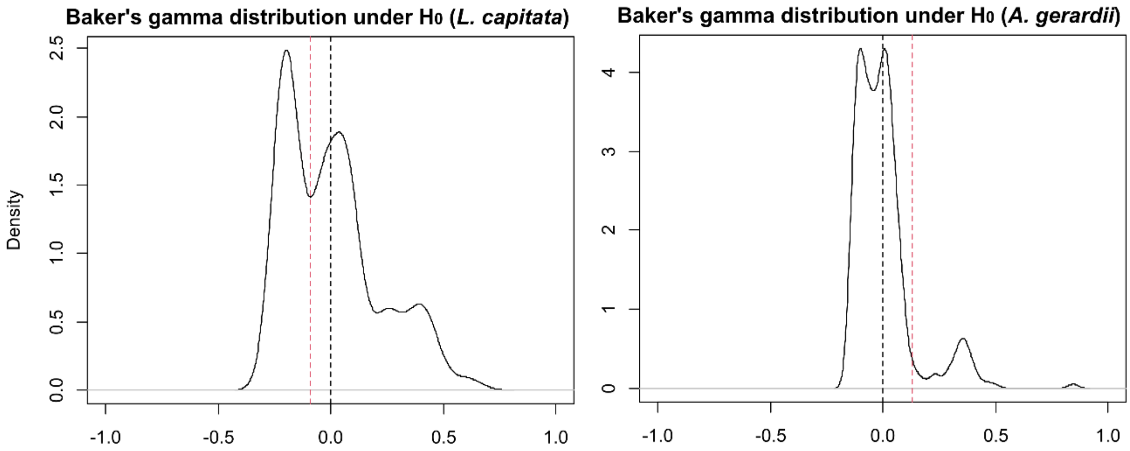

Figure S6: Distribution of Baker’s Gamma scores (solid black line) testing similarity of aqueous and lipid fraction sample clustering for 1000 random permutations of aqueous and lipid dendrograms. Dashed red line shows the score for the observed Baker’s Gamma for *L. capitata* (left) and *A. gerardi* (right), while dashed black line shows Baker’s Gamma of zero (no relationship between clustering of aqueous and lipid samples).

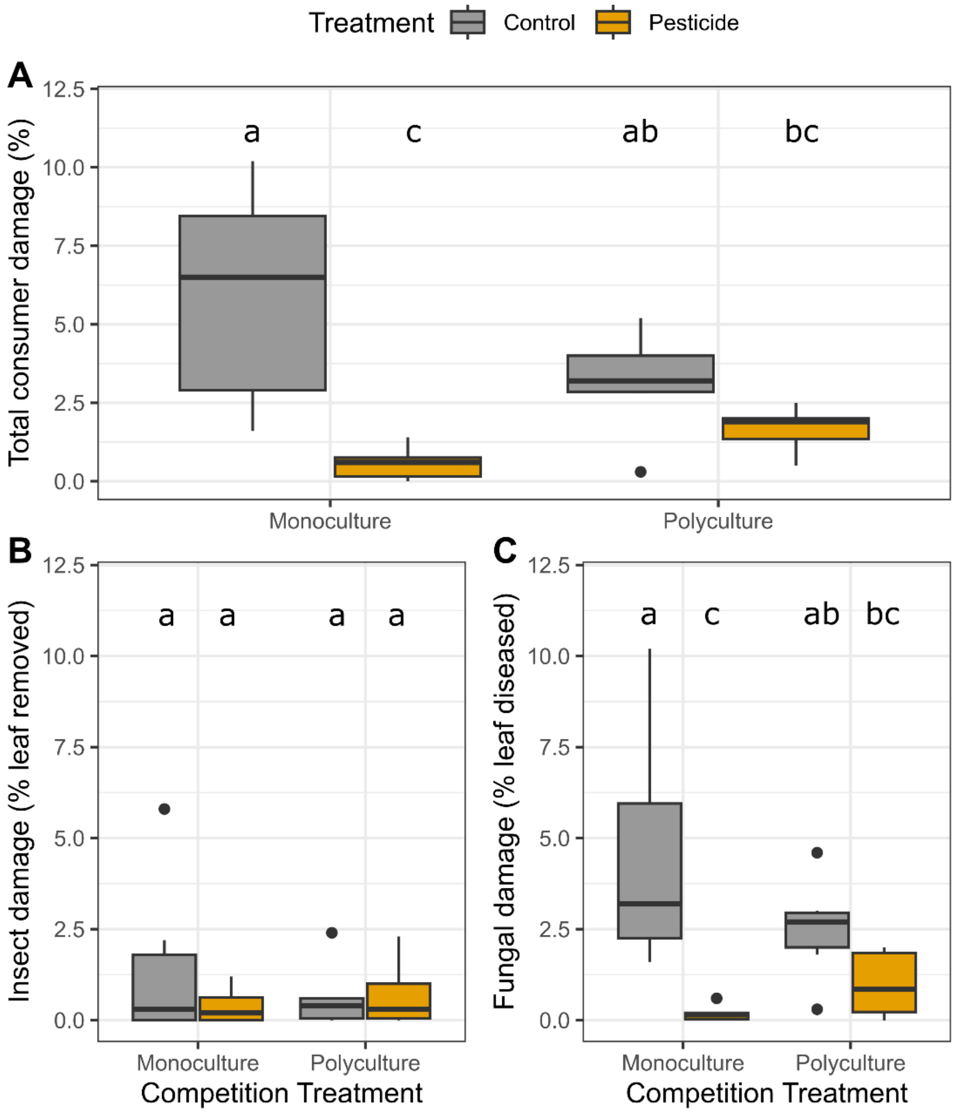

Figure S7: Percentage of leaf damage across consumer and competition treatments, for total insect and fungal damage combined (A), and insect damage (B) and fungal damage (C) separately. Letters above boxes indicate statistically different categories (Tukey's HSD) (computed separately for each panel).

Supporting Tables

Table S1: ^{1}H chemical shifts (δ, in ppm) of previously reported plant metabolites. Italics indicate compounds with multiple proton signals. Numbers indicate references (see bottom of table); letters indicate notes (see bottom of table); slashes (/) indicate minor differences in reported ppm between references. s=singlet; d=doublet; t=triplet; q=quartet; m=multiplet; dd=double doublet; dt=double triplet, etc; br_=broad (singlet, doublet etc.)

Chemical shift (δ)	Compound	Group
Aqueous extract		
0.78 (s)	*Steroids (general)*[1,3]	Steroid
0.79 (d)	*Cembratriene-4,6-diol*[1]	(Di)terpenoid
0.81 (d)	*Cembratriene-4,6-diol*[1]	(Di)terpenoid
0.85 (s)	*Steroids (general)*[1,3]	Steroid
0.86 (d)	*Capsidiol*[1]	(Sesqui)terpenoid
0.88 (t)	Fatty acids (general)[1,3]	Fatty acid
0.89 (d)	*Steroids (general)*[1,3]	Steroid
0.89 (s)	*Lupeol*[5]	(Tri)terpenoid
0.91 (d)	*Quercetin 3-O-rhamnoside*[5,7]	Flavonoid
0.91 (t)	Fatty acids (general)[9]	Fatty acid
0.93 (s)	β-*sitosterol*[5]	Steroid
0.94 (s)	*Lupeol*[5]	(Tri)terpenoid
0.94 (d)	*Stigmasterol*[5]	Steroid
0.94 (t)	Fatty acids (general)[9]	Fatty acid
0.96 (t)	α-*linolenic acids*[a,1,3]	Fatty acid
0.96 (s)	*Betulin*[5]	(Tri)terpenoid
0.97 (d)	*Leucine/isoleucine*[6]	Amino acid
0.98 (s)	*Betulin*[5]	(Tri)terpenoid
0.99 (d)	*Leucine/isoleucine*[6]	Amino acid
1.00 (s)	*Betulin*[5]	(Tri)terpenoid
1.00/1.01 (d)	*Valine*[3,8,9,10/5,6,7]	Amino acid
1.02 (s)	β-*sitosterol*[5]	Steroid
1.03 (s)	*Stigmasterol*[5]	Steroid
1.05/1.06/1.04 (d)	*Valine*[3,8,9,10/5,6/7]	Amino acid
1.07 (s)	*Lupeol*[5]	(Tri)terpenoid
1.10 (d)	Rhamnose in flavonoid[1,3,6]	Flavoinoid
1.19 (t)	1-*O*-Ethyl-β-d-glucoside[6,11]	Glycoside
1.30 (brs)	α-*linolenic acids*[a,1,3]	Fatty acid
1.30 (s)	*Monoacylmonogalactosylglycerol*[5]	Ester
1.32/1.34/1.33 (d)	*Threonine*[1,3,8,9/5/6,10]	Amino acid
1.33 (s)	*Capsidiol*[1]	(Sesqui)terpenoid
1.34 (m)	*Monoacylmonogalactosylglycerol*[5]	Ester
1.34/1.35 (m)	Fatty acids (general)[5/7]	Fatty acid
1.36 (d)	*Lactic acid*[9]	Phenolic acid
1.38 (s)	*Cembratriene-4,6-diol*[1]	(Di)terpenoid
1.48/1.39/1.49 (d)	*Alanine*[1,3,8,9,10/5/6,7]	Amino acid
1.70 (s)	*Cembratriene-4,6-diol*[1]	(Di)terpenoid
1.75 (s)	*Lupeol*[5]	(Tri)terpenoid
1.88/1.90/1.91 (m)	*GABA*[3/8,10/9]	Phenolic acid

1.91/1.90 (d)	*Caffeoylquinic acid*[5/7]	Phenolic acid
1.91 (s)	Acetate[10]	Ester
1.93/1.94 (s)	Acetic acid[6/9]	Phenolic acid
2.01 (m)	*Proline*[6]	Amino acid
2.04/2.03 (m)	*Caffeoylquinic acid*[b,1/6]	Phenolic acid
2.07/2.08/2.13 (m)	*Glutamate (glutamic acid)*[3,5,8/6/9,10]	Amino acid
2.12 (d)	Thujone[2]	(Mono)terpenoid
2.15/2.16/2.14 (m)	*Glutamine*[3,8/5/9,10]	Amino acid
2.17/2.15 (m)	*Caffeoylquinic acid*[b,1/6]	Phenolic acid
2.28/2.30/2.35 (t)	*GABA*[3/8,10/9]	Phenolic acid
2.28/2.30/2.29 (m)	*Valine*[6/9/10]	Amino acid
2.34 (m)	*Proline*[1,3,5,6,8]	Amino acid
2.36/2.35/2.42/2.46 (m)	*Glutamate (glutamic acid)*[3,8/5,6/9/10]	Amino acid
2.38 (dd)	*Malate*[10]	Ester
2.42 (dd)	*Malic acid*[e,6]	Phenolic acid
2.45/2.47/2.43 (m)	*Glutamine*[1/3,5,8,10/9]	Amino acid
2.54/2.45/2.56/2.53 (s)	Succinic acid[1,3/6/8/9]	Phenolic acid
2.56/2.53/2.54 (d)	*(Iso)citric acid*[1,3,8/5/6]	Phenolic acid
2.56 (dd)	*Catechin*[7]	Flavonoid
2.58 (dd)	*Malic acid*[d,e,9]	Phenolic acid
2.58-2.62 (dd)	*Malate (bound)*[10]	Ester
2.61 (dd)	*(Citra)malic acid*[1,3]	Phenolic acid
2.63 (dd)	*Caffeoylquinic acid*[b,7]	Phenolic acid
2.68 (dd)	*Malate*[10]	Ester
2.69/2.68 (dd)	*Malic acid*[e,6/8]	Phenolic acid
2.74/2.70/2.71 (d)	*Citric acid*[1,3,8/5/6]	Phenolic acid
2.74/2.76 (dd)	*Malic acid*[d,e,8/9]	Phenolic acid
2.77 (s)	*Nicotine*[1]	Alkaloid
2.80 (dd)	*(Citra)malic acid*[1,3]	Phenolic acid
2.82 (dd)	*Aspartic acid*[6]	Amino acid
2.83/2.84 (m)	*Catechin*[5/7]	Flavonoid
2.87 (dd)	*Progoitrin*[9]	Glucosinolate
2.96 (dd)	*Aspartic acid*[6]	Amino acid
3.01 (dd)	*GABA*[8,9,10]	Phenolic acid
3.04 (td)	*Isocitric acid*[3]	Phenolic acid
3.16 (dd)	*Histidine*[10]	Amino acid
3.20-3.93 (m)	*Schaftoside*[5]	Flavonoid
3.24 (t)	*Inositol*[1,3,8]	Sugar
3.24/3.22 (s)	Choline[5,7,8,9/6]	Building block (cation)
3.25/3.23 (d)	*IAA (Indoleacetic acid)*[9/11]	Phenolic acid (auxin)
3.25 (d)	*Histidine*[10]	Amino acid
3.28 (s)	Phosphatidylcholine[6]	Fatty acid
3.30 (d)	*Valine*[9]	Amino acid
3.28-3.92 (m)	*Orientin*[5]	Flavonoid
3.39/3.38 (d)	*IAA (Indoleacetic acid)*[9/11]	Phenolic acid (auxin)
3.40 (m)	α-*glucose*[10]	Sugar
3.41 (m)	*Proline*[6]	Amino acid
3.44 (dd)	*Inositol*[1,3,8]	Sugar

3.47 (dd)	α-*glucose*[10]	Sugar
3.51 (m)	*Monoacylmonogalactosylglycerol*[5]	Ester
3.52 (d)	*Threonine*[9]	Amino acid
3.58-3.62 (m)	*Gendarucin A*[5]	Flavonoid
3.61 (t)	*Inositol*[1,3,8]	Sugar
3.70-3.75 (m)	*Gendarucin A*[5]	Flavonoid
3.72 (t)	*Glutamate (glutamic acid)*[10]	Amino acid
3.72 (t)	*Glutamine*[1]	Amino acid
3.72 (dd)	*Monoacylmonogalactosylglycerol*[5]	Ester
3.72 (m)	*Ascorbic acid*[5]	Phenolic acid
3.73/3.78 (q)	*Alanine*[5,9,10/6]	Amino acid
3.75-3.81 (dd)	*Caffeoylquinic acid*[b,1,6]	Phenolic acid
3.76-4.85 (m)	*Vitexin*[5]	Flavonoid
3.89 (m)	*Monoacylmonogalactosylglycerol*[5]	Ester
3.95/3.93 (m)	*Catechin*[5/7]	Flavonoid
3.96 (dd)	*Aspartic acid*[6]	Amino acid
4.00 (t)	*Inositol*[1,3,8]	Sugar
4.00 (d)	*Isocitric acid*[3]	Phenolic acid
4.08 (dd)	*Proline*[1,3,6,8]	Amino acid
4.09-4.22 (td, brq)	*Caffeoylquinic acid*[b,1,7/6]	Phenolic acid
4.11/4.18/4.16 (d)	Fructose[1/5/9]	Sugar
4.14 (d)	*Lactic acid*[9]	Phenolic acid
4.17/4.18/4.16 (d)	*Sucrose*[1,3,8,10/6/9]	Sugar
4.23 (d)	*Monoacylmonogalactosylglycerol*[5]	Ester
4.23 (m)	*Threonine*[9]	Amino acid
4.28 (dd)	*Malate*[10]	Ester
4.32/4.28/4.34 (dd)	*Malic acid*[d,1,3,9/6/8]	Phenolic acid
4.33 (t)	*Capsidiol*[1]	(Sesqui)terpenoid
4.51 (dt)	*Capsidiol*[1]	(Sesqui)terpenoid
4.52/4.53 (d)	*Ascorbic acid*[1,3/5]	Phenolic acid
4.54 (s)	*Lupeol*[5]	(Tri)terpenoid
4.54 (d)	*Rutin*[5,7]	Glycoside
4.59/4.58 (d)	*β*-glucose[1,3,6,7,10/5,8,9]	Sugar
4.59/4.58 (d)	*Catechin*[5/7]	Flavonoid
4.61 (s)	*Betulin*[5]	(Tri)terpenoid
4.63 (m)	*Progoitrin*[9]	Glucosinolate
4.68 (s)	*Betulin*[5]	(Tri)terpenoid
4.68 (s)	*Lupeol*[5]	(Tri)terpenoid
4.73 (d)	*Capsidiol*[1]	(Sesqui)terpenoid
4.74 (d)	*Orientin*[5]	Flavonoid
4.74 (d)	*Schaftoside*[5]	Flavonoid
4.88 (s)	*Gendarucin A*[5]	Flavonoid
4.98 (m)	*Stigmasterol*[5]	Steroid
4.99/4.97 (d)	*Rutin*[5,7]	Glycoside
5.10 (m)	*Stigmasterol*[5]	Steroid
5.16 (d)	*Quercetin 3-O-glucoside*[7]	Flavonoid
5.18-5.21 (dd)	*Malate (bound)*[10]	Ester
5.20/5.19/5.18 (d)	α-*glucose*[1,3,6/5,7,10/8,9]	Sugar

5.21 (dt)	*Progoitrin*[9]	Glucosinolate
5.26 (d)	*Quercetin 3-O-arabinofuranoside*[7]	Flavonoid
5.33/5.34 (td)	*Caffeoylquinic acid*[b,1/6]	Phenolic acid
5.34 (dt)	*Progoitrin*[9]	Glucosinolate
5.40/5.43/5.42 (d)	*Sucrose*[1,3,7,8,10/5/6]	Sugar
5.47/5.49 (d)	*Quercetin 3-O-rhamnoside*[5/7]	Flavonoid
5.49 (td)	*(Di)caffeoylquinic acid*[6]	Phenolic acid
5.69 (s)	*Orientin*[5]	Flavonoid
5.93-5.99 (d)	*Malate (bound)*[9,10]	Ester
5.96 (dd)	*Capsidiol*[1]	(Sesqui)terpenoid
5.96 (m)	*Progoitrin*[9]	Glucosinolate
6.18 (d)	*Quercetin 3-O-arabinofuranoside*[7]	Flavonoid
6.18 (d)	*Quercetin 3-O-rhamnoside*[7]	Flavonoid
6.20 (d)	*Rutin*[7]	Glycoside
6.20 (d)	*Quercetin*[7]	Flavonoid
6.20 (d)	*Quercetin 3-O-glucoside*[7]	Flavonoid
6.28/6.30 (d)	*Quercetin*[c,1,3/6]	Flavonoid
6.28 (d)	*Kaempferol*[1,3]	Flavonoid
6.29/6.31 (d)	*Caffeic acid*[3/6]	Phenolic acid
6.33 (d)	*Ferulic acid*[3]	Phenolic acid
6.35 (d)	*Quercetin 3-O-rhamnoside*[7]	Flavonoid
6.36-6.41 (d)	*Caffeoylquinic acid*[b,1,6,7]	Phenolic acid
6.37 (?)	*Sinapic acid*[3]	Phenolic acid
6.39/6.37 (d)	*Quercetin 3-O-arabinofuranoside*[5/7]	Flavonoid
6.39 (d)	*Quercetin 3-O-glucoside*[7]	Flavonoid
6.39 (d)	*Rutin*[5,7]	Glycoside
6.42/6.40 (d)	*Quercetin*[5,7]	Flavonoid
6.43-6.50 (d)	*Malate (bound)*[9,10]	Ester
6.46 (d)	*Kaempferol analogues*[9]	Flavonoid
6.46 (d)	*Catechin*[7]	Flavonoid
6.47 (d)	*Quercetin analogues*[9]	Flavonoid
6.48 (d)	*(Di)caffeoylquinic acid*[6]	Phenolic acid
6.49 (d)	*Catechin*[7]	Flavonoid
6.52/6.50 (d)	*Quercetin*[c,1,3/6]	Flavonoid
6.52 (d)	*Kaempferol*[1,3]	Flavonoid
6.53 (s)	Fumarate[10]	Ester
6.56/6.54 (s)	Fumaric acid[3,5,8,9/6]	Phenolic acid
6.58 (d)	*Orientin*[5]	Flavonoid
6.69 (s)	*Isoorientin*[5]	Flavonoid
6.74 (d)	*Kaempferol*[1,3]	Flavonoid
6.77 (s)	*(Iso)vitexin*[5]	Flavonoid
6.77 (d)	*Kaempferol analogues*[9]	Flavonoid
6.77 (d)	*Quercetin analogues*[9]	Flavonoid
6.80 (s)	*Schaftoside*[5]	Flavonoid
6.80-6.87 (d)	*Malate (bound)*[9,10]	Ester
6.85/6.83 (d)	*Quercetin*[5,7]	Flavonoid
6.86 (t)	*Tyrosine*[3,8]	Amino acid
6.86 (d)	*Quercetin 3-O-glucoside*[7]	Flavonoid

6.87 (s)	*Isovitexin*[5]	Flavonoid
6.87-6.89 (d)	*Caffeoylquinic acid*[b,1,6]	Phenolic acid
6.88/6.89 (d)	*Caffeic acid*[3/6]	Phenolic acid
6.88 (d)	*Ferulic acid*[3]	Phenolic acid
6.89/6.88 (d)	*Quercetin 3-O-rhamnoside*[5/7]	Flavonoid
6.89/6.88 (d)	*Quercetin 3-O-arabinofuranoside*[5/7]	Flavonoid
6.90 (d)	*Isoorientin*[5]	Flavonoid
6.90 (d)	*Schaftoside*[5]	Flavonoid
6.90 (d)	*Gendarucin A*[5]	Flavonoid
6.90 (d)	*Quercetin analogues*[9]	Flavonoid
6.90-6.95 (d, s)	*Malate (bound)*[9,10]	Ester
6.91 (d)	*(Iso)vitexin*[5]	Flavonoid
6.91/6.90 (d)	*Rutin*[5,7]	Glycoside
6.92/6.94 (d)	p-*hydroxy benzoic acid*[3/6]	Phenolic acid
6.93 (s)	*Sinapic acid*[3]	Phenolic acid
6.94 (s)	*Gendarucin A*[5]	Flavonoid
6.99/6.98 (d)	*Quercetin*[c,1,3/6]	Flavonoid
6.99 (d)	*Gendarucin A*[5]	Flavonoid
7.00 (d)	*Kaempferol analogues*[9]	Flavonoid
7.00 (bd)	*Malate (bound)*[9]	Ester
7.03/7.07 (d/dd)	*Caffeic acid*[3/6]	Phenolic acid
7.05/7.07 (d/dd)	*Caffeoylquinic acid*[b,1/6]	Phenolic acid
7.06 (dd)	*Malate (bound)*[9]	Ester
7.08 (s)	Gallic acid[5]	Phenolic acid
7.10 (d)	*Ferulic acid*[3]	Phenolic acid
7.12 (d)	*Histidine*[10]	Amino acid
7.12/7.15 (d)	*Caffeic acid*[3/6]	Phenolic acid
7.12 (s)	*IAA (Indoleacetic acid)*[9,11]	Phenolic acid (auxin)
7.13/7.10 (t)	*IAA (Indoleacetic acid)*[9/11]	Phenolic acid (auxin)
7.13-7.15 (dd)	*Malate (bound)*[9,10]	Ester
7.15 (d)	*Caffeoylquinic acid*[b,1,6]	Phenolic acid
7.19 (d)	*Ferulic acid*[3]	Phenolic acid
7.19 (t)	*Tyrosine*[3,8]	Amino acid
7.20 (t)	*Tryptophan*[3,8]	Amino acid
7.21/7.27 (t)	*IAA (Indoleacetic acid)*[9/11]	Phenolic acid (auxin)
7.22 (s)	*Orientin*[5]	Flavonoid
7.26 (d)	*Malate (bound)*[9]	Ester
7.29 (t)	*Tryptophan*[3,8]	Amino acid
7.30 (dd)	*Quercetin 3-O-rhamnoside*[7]	Flavonoid
7.33 (d)	*Orientin*[5]	Flavonoid
7.32/7.34 (s)	*Tryptophan*[3,8/5]	Amino acid
7.39 (d)	*Isoorientin*[5]	Flavonoid
7.41 (s)	*Isoorientin*[5]	Flavonoid
7.47 (dd)	*Quercetin 3-O-arabinofuranoside*[7]	Flavonoid
7.47/7.45 (d)	*IAA (Indoleacetic acid)*[9/11]	Phenolic acid (auxin)
7.48 (?)	*Sinapic acid*[3]	Phenolic acid
7.51 (d)	*Malate (bound)*[9]	Ester
7.52/7.57 (d)	*Caffeic acid*[3/6]	Phenolic acid

7.54 (d)	*Tryptophan*[3,8]	Amino acid
7.54 (dd)	*Quercetin analogues*[9]	Flavonoid
7.56 (d)	*Ferulic acid*[3]	Phenolic acid
7.56 (dd)	*Rutin*[5,7]	Glycoside
7.58-7.67 (d)	*Malate (bound)*[9,10]	Ester
7.59 (d)	*IAA (Indoleacetic acid)*[11]	Phenolic acid (auxin)
7.60/7.61/7.64/7.62 (d)	*Caffeoylquinic acid*[b,1/5/6/7]	Phenolic acid
7.60 (dd)	*Quercetin 3-O-glucoside*[7]	Flavonoid
7.62/7.60 (dd)	*Quercetin*[c,6/7]	Flavonoid
7.72 (d)	*IAA (Indoleacetic acid)*[9]	Phenolic acid (auxin)
7.73/7.72 (d)	*Tryptophan*[3,8/5]	Amino acid
7.73 (d)	*Quercetin*[5,7]	Flavonoid
7.73 (d)	*Quercetin 3-O-rhamnoside*[5]	Flavonoid
7.73 (d)	*Quercetin 3-O-arabinofuranoside*[5,7]	Flavonoid
7.73 (d)	*Quercetin 3-O-glucoside*[7]	Flavonoid
7.82 (d)	*Quercetin analogues*[9]	Flavonoid
7.89 (bd)	*Malate (bound)*[9]	Ester
7.90 (d)	*Isovitexin*[5]	Flavonoid
7.91/7.92 (d)	p-*hydroxy benzoic acid*[3/6]	Phenolic acid
7.93 (d)	*Histidine*[10]	Amino acid
7.98 (d)	*Schaftoside*[5]	Flavonoid
8.00 (d)	*Vitexin*[5]	Flavonoid
8.04 (d)	*Kaempferol*[1,3]	Flavonoid
8.07 (d)	*Kaempferol analogues*[9]	Flavonoid
8.10 (dt)	*Nicotine*[1]	Alkaloid
8.19/8.20 (s)	*Adenine*[5,6/8,9]	Building block (cytokinin)
8.21 (s)	*Adenine*[5,6,8,9]	Building block (cytokinin)
8.21 (s)	*Gendarucin A*[5]	Flavonoid
8.47/8.48/8.46 (s)	Formic acid[5,7/6/8,9]	Phenolic acid
8.66 (dd)	*Nicotine*[1]	Alkaloid
8.70 (d)	*Nicotine*[1]	Alkaloid
Lipid extract		
0.88-0.98	-C$\underline{H}_3$ protons[4]	
2.01-2.46	Allylic protons[4] -C$\underline{H}_2$-	
2.78	Bis-allyic protons[4] =HC–C$\underline{H}_2$–CH= (linoleic acid)	
2.82	Bis-allyic protons[4] =HC–C$\underline{H}_2$–CH= (α-linoleic acid)	
2.85	Bis-allyic protons[4] =HC–C$\underline{H}_2$–CH= (DHA)	
2.99-3.07	Methylene (CH_2) group in esters[4]	
3.59	Glycerol (monoacylglycerides)[4]	
3.72	*Diacylglyceride protons*[4]	
4.03	*Diacylglyceride protons*[4]	
4.13	*Diacylglyceride protons*[4]	
4.15	*Glycerol (triglycerides)*[4]	
4.18	*Diacylglyceride protons*[4]	
4.23	*Diacylglyceride protons*[4]	
4.30	*Glycerol (triglycerides)*[4]	
4.31	*Diacylglyceride protons*[4]	
5.08	*Diacylglyceride protons*[4]	

5.20-5.50	Olefinic protons[4] –CH=CH–
5.26	Glycerol backbone (triglycerides)[4]
5.99-6.85	Oleofinic protons of conjugated linoleic acids[4] –C<u>H</u>=

Notes:

[a] Precursor to jasmonate, but also potentially a direct defence chemical[1]

[b] Also known as chlorogenic acid. This and other phenylpropanoids act as constitutive phytoanticipins[1]. Proton shifts largely consistent among references, but some addition shifts reported from reference 6.

[c] Quercetin analogue, could also be luteolin analogue[6]

[d] These shifts are for free malic acid; additional shifts for malic acid bound to phenylpropanoids include 2.65-2.70, 2.83-2.85, 5.19-5.22

[e] Chemical shift of malic acid in this region changeable with pH and concentration

References:

1. Choi, Y. H., Kim, H. K., Linthorst, H. J., Hollander, J. G., Lefeber, A. W., Erkelens, C., ... & Verpoorte, R. (2006). NMR metabolomics to revisit the tobacco mosaic virus infection in *Nicotiana tabacum* leaves. Journal of Natural Products, 69(5), 742-748.
2. Monakhova, Y. B., Kuballa, T., & Lachenmeier, D. W. (2011). Rapid determination of total thujone in absinthe using 1H NMR spectroscopy. International Journal of Spectroscopy, 2011, 171684.
3. Verpoorte, R., Choi, Y. H., & Kim, H. K. (2007). NMR-based metabolomics at work in phytochemistry. Phytochemistry reviews, 6, 3-14.
4. Alexandri, E., Ahmed, R., Siddiqui, H., Choudhary, M. I., Tsiafoulis, C. G., & Gerothanassis, I. P. (2017). High resolution NMR spectroscopy as a structural and analytical tool for unsaturated lipids in solution. Molecules, 22(10), 1663.
5. Khoo, L. W., Mediani, A., Zolkeflee, N. K. Z., Leong, S. W., Ismail, I. S., Khatib, A., ... & Abas, F. (2015). Phytochemical diversity of *Clinacanthus nutans* extracts and their bioactivity correlations elucidated by NMR based metabolomics. Phytochemistry Letters, 14, 123-133.
6. Liu, N. Q., Cao, M., Frédérich, M., Choi, Y. H., Verpoorte, R., & van der Kooy, F. (2010). Metabolomic investigation of the ethnopharmacological use of *Artemisia afra* with NMR spectroscopy and multivariate data analysis. Journal of ethnopharmacology, 128(1), 230-235.
7. Mediani, A., Abas, F., Khatib, A., Maulidiani, H., Shaari, K., Choi, Y. H., & Lajis, N. H. (2012). 1H-NMR-based metabolomics approach to understanding the drying effects on the phytochemicals in *Cosmos caudatus*. Food Research International, 49(2), 763-770.
8. Kim, H. K., Choi, Y. H., & Verpoorte, R. (2010). NMR-based metabolomic analysis of plants. Nature protocols, 5(3), 536-549.
9. Abdel-Farid, I. B., Kim, H. K., Choi, Y. H., & Verpoorte, R. (2007). Metabolic characterization of *Brassica rapa* leaves by NMR spectroscopy. Journal of Agricultural and Food Chemistry, 55(19), 7936-7943.
10. Jahangir, M., Kim, H. K., Choi, Y. H., & Verpoorte, R. (2008). Metabolomic response of *Brassica rapa* submitted to pre-harvest bacterial contamination. Food Chemistry, 107(1), 362-368.
11. Liang, Y. S., Choi, Y. H., Kim, H. K., Linthorst, H. J., & Verpoorte, R. (2006). Metabolomic analysis of methyl jasmonate treated *Brassica rapa* leaves by 2-dimensional NMR spectroscopy. Phytochemistry, 67(22), 2503-2511.

Table S2: All modules and their associated chemical shifts from the weighted network analysis for (a) overall phytochemical composition across both species, (b) *L. capitata*, and (c) *A. gerardi*. The contribution of each module to the principal component analyses are shown in Figs. S1 – S3 respectively.

**Module**	**Chemical shifts (δ) in 0.05 ppm bins**	**Potential associated chemical classes (through comparison with Table S1)**
(a) Overall analysis		
1	0.70-0.75, 0.85, 1.40, 1.90-2.45, 2.55, 2.65, 2.80, 3.00, 3.15, 3.55, 3.70, 3.85-3.90, 4.05-4.20, 5.15, 5.45, 5.80-6.25, 6.35, 6.55-6.60, 7.00, 7.10, 7.25, 7.80	Largely signals of simple phenolic acids (<5.80), with signals >5.80 generally falling in the flavonoid zone
2	0.80, 2.85, 3.10, 3.65, 4.00, 4.40-4.65, 5.25, 6.80-6.95, 7.15	General module of with signals of sugars, terpenoids and some phenolic acids
3	5.00, 5.35, 6.40, 7.20, 7.65-7.70	Glycoside signals (reasonable match to rutin), some amino acid signals
4	2.95, 6.45, 7.35-7.40	No consistent signals
5	0.65, 4.35, 6.75, 7.60	Signals match possible esters
6	0.55-0.60, 0.90-1.05, 1.25-1.35, 1.45-1.65, 1.80-1.85, 2.50, 2.60, 2.75, 2.90, 3.05, 3.20, 3.50, 3.75-3.80, 3.95, 4.25-4.30, 4.95, 5.05-5.10, 5.30, 5.40, 5.50-5.75, 6.30, 6.50, 6.65-6.70, 7.05, 7.30, 7.45-7.55, 7.75, 7.85-9.50	A ‘catch-all’ module consisting of signals that do not contribute strongly to either PC1 or PC2, with low correlation weights and high noise
7	1.10-1.20, 1.70-1.75, 3.60, 5.20	Tri- and di-terpenoids (early shifts), plus potential signals of sugars (inositol and α-glucose)
(b) *L. capitata*		
1	1.60-1.70, 1.85, 2.25, 3.00	Terpenoids and phenolic acids
2	1.05-1.55, 1.80, 2.15-2.20, 2.30, 3.05, 3.60, 5.05, 5.40, 7.60-7.65, 8.05-8.10	Terpenoids, fatty acids and steroids, some phenolic acid signals
3	2.00, 2.45-2.50, 2.75, 2.90, 3.20, 5.00, 6.30, 6.40, 6.50, 6.60-6.65, 6.95, 7.40, 7.70, 7.85	Consistent amino and phenolic acid signals
4	3.50, 3.70, 3.85-3.90, 4.05, 4.20, 5.10, 5.45	Clear sugar module; correspondence with sucrose, α-glucose and inositol
5	3.55, 3.65, 3.75-3.80, 4.00	Signals match phenolic acids, correspondence with gendarucin A
6	3.95, 4.25, 5.30, 5.35, 7.25-7.30	Amino acid signals, correspondence with threonine and tryptophan
7	0.80, 2.10, 5.55, 7.00, 7.45, 7.90	No consistent signals
8	1.75, 4.60-4.65, 5.20-5.25	Di- and tri-terpenoid signals
9	2.95, 5.15, 5.85, 6.05, 6.15-6.25, 6.45, 6.55, 7.35, 7.80	Generally matches flavonoid signals, correspondence with quercetin and its analogues
10	6.55, 7.35, 7.80	General flavonoid signals
11	0.55-0.75, 1.90-1.95, 2.05, 2.35-2.40, 2.55-2.70, 2.80, 3.15, 4.10-4.15, 4.35-	A ‘catch-all’ module consisting of signals that do not contribute

	4.55, 4.95, 5.50-5.75, 6.70, 7.05-7.20, 7.50-7.55, 7.75, 7.95, 8.15-9.50	strongly to either PC1 or PC2, with low correlation weights and high noise
12	2.85, 3.10, 5.80, 5.90-6.00, 6.10, 6.35, 6.75-6.90, 8.00	No consistent signals
(c) *A. gerardi*		
1	2.70, 4.30-4.35, 4.55	Strong correspondence with malate and malic acid
2	1.05-1.25, 1.45, 1.55-1.60, 1.70-1.80, 2.75, 4.45-4.50, 5.00-5.05, 5.40, 6.35, 6.55, 7.70, 7.95	Signals of terpenoids and phenolic acids
3	2.00, 3.70-3.85, 4.05, 4.20, 5.45, 7.60	Amino acids and sugars (strong correspondence with sucrose)
4	2.15-2.20, 2.30, 2.45, 2.65	Phenolic acids
5	0.70-0.75, 3.05-3.15, 4.15, 4.60, 5.15, 6.45, 6.60, 7.15, 7.35-7.40	Signals of sugars (glucose, fructose), some general flavonoid shifts
6	4.65, 5.20-5.25	No consistent signals
7	1.90-1.95, 2.05	Phenolic acids; correspondence with caffeoylquinic acid
8	2.80-2.85, 2.95-3.00	Amino acids; correspondence with aspartic acid
9	0.55-0.65, 0.90-1.00, 1.30-1.40, 1.65, 1.85, 2.10, 2.25, 2.35-2.40, 2.50-2.60, 2.90, 3.20, 3.50-3.65, 3.90-4.00, 4.10, 4.25, 4.40, 4.95, 5.10, 5.30, 5.50-6.30, 6.50, 6.65-7.05, 7.25-7.30, 7.45-7.55, 7.75-7.90, 8.00-9.50	A ‘catch-all’ module consisting of signals that do not contribute strongly to either PC1 or PC2, with low correlation weights and high noise
10	0.80, 5.35, 6.40, 7.10, 7.20, 7.65	No consistent signals
